# Contribution of neuronal calcium sensor 1 (Ncs-1) to anxiolytic-like and social behavior mediated by valproate and Gsk3 inhibition

**DOI:** 10.1038/s41598-020-61248-z

**Published:** 2020-03-12

**Authors:** Luiz Alexandre Viana Magno, Helia Tenza-Ferrer, Mélcar Collodetti, Eduardo de Souza Nicolau, Jivan Khlghatyan, Thomas Del’Guidice, Marco Aurélio Romano-Silva, Jean Martin Beaulieu

**Affiliations:** 1 Centro de Tecnologia em Medicina Molecular, Belo Horizonte, Brazil; 2 Departamento de Saúde Mental, Faculdade de Medicina, Universidade Federal de Minas Gerais (UFMG), Belo Horizonte, CEP 30130-100 Brazil; 3 Department of Psychiatry and Neuroscience, Laval University, Québec, Canada; 4 Department of Pharmacology & Toxicology, University of Toronto, Toronto, Canada; 5 Present Address: Feldan Therapeutics, Québec City, Canada

**Keywords:** Cellular neuroscience, Molecular neuroscience, Social behaviour

## Abstract

Peripheral biomarker and post-mortem brains studies have shown alterations of neuronal calcium sensor 1 (Ncs-1) expression in people with bipolar disorder or schizophrenia. However, its engagement by psychiatric medications and potential contribution to behavioral regulation remains elusive. We investigated the effect on Ncs-1 expression of valproic acid (VPA), a mood stabilizer used for the management of bipolar disorder. Treatment with VPA induced Ncs-1 gene expression in cell line while chronic administration of this drug to mice increased both Ncs-1 protein and mRNA levels in the mouse frontal cortex. Inhibition of histone deacetylases (HDACs), a known biochemical effect of VPA, did not alter the expression of Ncs-1. In contrast, pharmacological inhibition or genetic downregulation of glycogen synthase kinase 3β (Gsk3β) increased Ncs-1 expression, whereas overexpression of a constitutively active Gsk3β had the opposite effect. Moreover, adeno-associated virus-mediated Ncs-1 overexpression in mouse frontal cortex caused responses similar to those elicited by VPA or lithium in tests evaluating social and mood-related behaviors. These findings indicate that VPA increases frontal cortex Ncs-1 gene expression as a result of Gsk3 inhibition. Furthermore, behavioral changes induced by Ncs-1 overexpression support a contribution of this mechanism in the regulation of behavior by VPA and potentially other psychoactive medications inhibiting Gsk3 activity.

## Introduction

The neuronal calcium sensor 1 (Ncs-1) is a Ca^2+^-binding protein that regulates neurotransmitter release^1^, dopamine D2 receptor (D2R) desensitization^2^ and neuronal survival^3^, among other functions^4,5^. Alterations in Ncs-1 levels may contribute to psychiatric disorders. Indeed, the expression pattern of Ncs-1 is altered in schizophrenia and bipolar disorder patients^6,7^. In addition, inducible overexpression of Ncs-1 in the brain of rodents enhances exploration and spatial memory acquisition, and increases axonal sprouting^8,9^, whereas loss of Ncs-1 in knockout (KO) mice caused depressive-like, anxiety-like and impaired motivated behaviors^10,11^.

Although evidence suggests that Ncs-1 is affected in schizophrenia and bipolar disorder, the effect of psychiatric medications on Ncs-1 remains unexplored. We investigated whether the mood-stabilizing drug valproate (VPA) could contribute to the regulation of brain Ncs-1. VPA is a first-line treatment for depressive and manic phases of bipolar disorder^12^. It alters gene expression and promotes neuroplasticity changes, which in recent years has been suggested to underlie malfunctioning of the neurocircuits related to the psychiatric symptoms^13–15^. Inhibition of histone deacetylases (HDACs) and glycogen synthase kinase 3 (Gsk3α and β) are the most prominent mechanisms suspected to be involved in the mood-stabilizing effects of VPA^16–19^.

VPA has been demonstrated to be an inhibitor of HDACs both *in vitro* and *in vivo*^20,21^. Chromatin remodeling mediated through HDAC inhibition may lead to transcriptional control of target genes related to neuronal differentiation, protection, and the regulation of behavioral dimensions such as mood and cognitive states^16,17,22^.

Inhibition of brain Gsk3 activity is a shared effect of several psychoactive drugs including VPA and lithium and strong evidence support a role for Gsk3 inhibition in the regulation of behavior by these drugs^23,24^. Gsk3 can regulate gene expression by acting on transcription factors such as β-catenin and Creb^25,26^. Furthermore, Gsk3 activity can also modulate mRNA translation by regulating mTor signaling and the activity of RNA binding proteins such as fragile X autosomal homolog 1 (Fxr1)^19,27,28^. VPA targets multiple neuronal processes mediated by Gsk3 and appear to depend on Gsk3 signaling to achieve behavioral regulation in preclinical animal models^18,19,29,30^.

The mechanism by which VPA inhibits Gsk3 remains partially characterized. On the one hand, VPA is not a direct inhibitor of Gsk3α or Gsk3β^31^. On the other hand, administration of VPA in mice has been shown to lead to an activation of brain Akt^19^, which in turns phosphorylates Gsk3α and Gsk3β leading to their inhibition^32^. The modulation of Akt and Gsk3 activity by VPA does not happen in mice lacking the signaling adaptor molecule beta-arrestin-2 (βArr2), which mediates both G-protein coupled receptor (GPCR) desensitization and signaling^19,33^. Furthermore, there are evidence that the D2R, which is a GPCR that signals via βArr2 to inactivate Akt^32^, also plays a role in the regulation of Gsk3 by VPA^19^.

Given that array of cellular effects, VPA treatment causes several unwanted side effects^34^. It is critical to identify molecular targets that mediate behavioral responses to VPA. We examined the impact of VPA administration on Ncs-1 expression in cells and in the mouse brain. Our findings uncovered that VPA upregulates Ncs-1. This effect is mediated by the inhibitory action of VPA on Gsk3, which results in significant increase of Ncs-1 mRNA and protein levels in both cell lines and the mouse frontal cortex. Furthermore, upregulation of Ncs-1 in the dorsomedial frontal cortex was sufficient to promote anxiolytic-like and pro-social behaviors in mice.

## Methods

### DNA constructs and AAVs preparation

The pGsk3β-S9A, pCMV-βgal, GFP and GFP-Fxr1 plasmids were previously described^29,35–37^. The promoterless pGL4.10[luc2] vector was obtained elsewhere (Promega). The upstream 2 kb genomic sequence of the human *NCS-1* gene was cloned into the pGL4.10[luc2] to create pGL4.10-NCS1.2005 (2,005 bp). Subsequent deletion constructs were created by PCR amplifying smaller fragments from this genomic sequence (pGL4.10-NCS1.1048 and pGL4.10-NCS1.588 containing 1,048 bp and 588 bp, respectively). Negative control plasmids pGL4.10-CKAMP44.2063 (2,063 bp), pGL4.10-CKAMP44.1017 (1,017 bp) and pGL4.10-CKAMP44.614 (614 bp) were constructed from exon 5 of the human *CKAMP44* gene. *CKAMP44* inserts display no known or predicted promoter activity. Supplementary Table S1 shows the genomic regions and all primers used for the creation of constructs. All sequences were confirmed by DNA sequencing. The full-length Ncs-1 mouse cDNA was inserted into the pAAV-hSyn-hChR2(H134R)-EYFP plasmid in the place of the hChR2(H134R), forming AAV/hSyn-NCS1-EYFP. Adenoassociated viruses (AAV) serotype 5 for AAV/hSyn-EYFP and AAV/hSyn-NCS1-EYFP were produced by the University of North Carolina Vector Core.

### *In vitro* assays

PC12, HEK-293 or Neuro-2A (N2A) cells were cultured in 6-well plates at 37 °C in a humidified incubator with 5% CO_2_ in DMEM high glucose supplemented with penicillin/streptomycin, heat-inactivated bovine serum (10% for HEK-293 and N2A, and 5% for PC12) and 5% fetal bovine (for PC12 only). Cells were seeded in triplicate for 48 h and cell confluence was strictly kept between 70–80% before drug administration. Cells were treated with 0.625 mM valproate sodium salt (VPA; Sigma-Aldrich #P4543), 10 μM TDZD-8 (Sigma-Aldrich #8325), 10 μg/mL cycloheximide (CHX; Sigma-Aldrich #01810) or 2.5–10 mM SAHA (Sigma-Aldrich #SML0061) at the indicated times in the result section. Compounds were freshly prepared in DMEM or DMSO before each series of experiment. The MTT reagent (Sigma-Aldrich #M5655) and Hoechst 33342 staining (Sigma-Aldrich #14533) were used to determine cell viability. N2A cells were transfected with 500 ng of GFP or GFP-Fxr1 plasmids using JetPrime reagent (Polypus Transfection) according to the manufacturer’s protocol. Transfections of PC12 and HEK-293 cells were performed by electroporation. Transfected cells were incubated for 48 hours before the experimental studies. Reporter assays were carried out in cells co-transfected with pGL4.10[luc2] vectors (experimental reporter) and pCMV-βgal. Luciferase and galactosidase activity from at least five different transfections carried out in triplicate were determined using a dual chemiluminescence detection kit (NovaBright™). Data are presented as β-Gal-normalized luciferase activity of pGL4.10-NCS1 cells relative to pGL4.10-CKAMP44 cells (RLUC).

### Animals and drug administration

C57Bl6/J were obtained from the Jackson Laboratory. βArr2, D2R and CamKIICre-Gsk3β^Flox/Flox^ mice were described previously^32,38^. In all experiments, respective WT littermates were used as controls for mutant mice. Experiments were performed with adult male mice housed in plastic cages in a humidity-controlled facility maintained on a 12-h light/dark cycle (lights on at 7:00 a.m.). All animals were kept with food and water available *ad libitum* throughout the experiments. For acute treatments, VPA (400 mg/kg i.p.) was dissolved in saline (0.9% NaCl) and samples collected 3 h after injection. The Gsk3β inhibitor TDZD-8 (30 mg/kg i.p.) was injected after suspension in a minimal amount of tween and brought to volume with distilled water as described previously^39^. Brain samples from the TDZD-8 treatment were collected 1 h after injection. Valproate chronic treatment lasted for 21 days and was performed as described previously^29^. Briefly, mice were divided into two groups: one group received standard chow and the other had VPA added to the food at 25 g of drug per 1 kg of chow. MS-275 (20 mg/kg i.p. daily for 21 days; Sigma-Aldrich #EPS002) was injected after dissolved in a restricted minimal volume of DMSO and brought to the final concentration with distilled water. In chronic treatments, mice were killed 4 h after the last drug administration. Brain structures were dissected, rapidly frozen and stored at −80 °C until assayed. The Laval University Institutional Animal Care Committee approved all animal experimental procedures according to guidelines from the Canadian Council on Animal Care (CCAC).

### Stereotaxic Injections

Stereotaxic injections were performed as previously described^40^. After performing a craniotomy, 1.0 μL of AAV/hSyn-NCS1-EYFP (4 ×10^12^ vg/mL), AAV/hSyn-EYFP (4 ×10^12^ vg/mL), GFP-Fxr1 (4.4 ×10^12^ vg/mL) or GFP (3 ×10^12^ vg/mL), was injected per site (anterior-posterior (AP), +2.4 mm; medio-lateral (ML), ±0.5 mm; dorso-ventral (DV), 1.7 mm for dmPFC) using a microinjector at 0.1 μL/min. All measures were taken before, during, and after surgery to minimize animal pain and discomfort. In all cases, sexual naïve male mice were randomly assigned into the experimental groups and injected at 8 weeks of age.

### Synaptosome isolation

Synaptosomes were isolated using Syn-PER reagent according to manufacturer’s recommendations (Thermo Fisher Scientific).

### Western blotting

Western blot was performed as previously described^29^. Aliquots containing equal amounts of protein (25 µg) from each sample were loaded onto a 10% Nupage Bis-Tris gel (Thermo Fisher Scientific), and then subjected to electrophoresis. After separation, proteins were transferred to a nitrocellulose membrane and incubated with blocking buffer (4% bovine serum albumin) for 1 h at room temperature. Blots were probed overnight using primary antibodies against Ncs-1 (1:1,000, RRID:AB_649907), Gsk3α/β (1:1,000, RRID:AB_10547140), anti-Actin (1:10,000, RRID:AB_2223041), anti-GAPDH (1:5,000, RRID:AB_627679), anti-Fxr1 (1:1,000, RRID:AB_11154960) and anti-GFP (1:1,000, RRID:AB_218216). Then we performed incubation with secondary antibodies (1:10,000) conjugated with horseradish peroxidase: anti-mouse (RRID:AB_257867), anti-rabbit (RRID:AB_11010558) and anti-goat (1:10,000, Arigo #ARG23857); or infrared fluorescence: anti-mouse IRDye680 (RRID:AB_10706161), anti-rabbit IRDye800 (RRID:AB_621843). Quantitative analyses of chemiluminescence or infrared signals were carried out using ImageJ^41^ and Image Studio Lite 5.2 (LI-COR), respectively. Western blot quantifications were normalized against β-actin, β-tubulin or GADPH and plotted relative to vehicle-treated samples, which was set to 1. All full blots are available in Supplementary Information file.

### RNA isolation and qPCR

Total RNA was obtained by using the Trizol reagent. Purified samples containing 500 ng of total RNA were reverse transcribed into cDNA. qPCR was performed using iQ^™^ SYBR Green (Bio-Rad) with the primers shown in Table S2. The fold change in *NCS-1* mRNA levels was relative to matched vehicle-treated controls and calculated after adjusting for *β-ACTIN* using the 2^−ΔΔCt^ method.

### Histology

Histology was performed as previously described^40^. Tissue sections were incubated with a chicken polyclonal antibody against NeuN (1:1,000, Millipore #ABN91, RRID:AB_11212808) and then labelled with a goat anti-chicken Alexa 594 secondary antibody (1:10,000, Thermo #A-11042, RRID:AB_253409). Three-dimensional z-stacks were acquired using a Leica SP5 confocal microscope.

### Behavioral measurements

Open field was performed to assess horizontal activity in open field boxes (40 cm long, 40 cm wide and 50 cm deep) as previously described^40^. Mice were allowed to explore the apparatus for 5 minutes. For elevated plus maze (EPM), mice were gently placed in the periphery of the closed arms and the session lasted 5 min. Time and number of entries in open arms were used for analysis. The dark-light emergence test (DLET) was performed for a period of 5 min with mice placed initially at the center of the dark chamber as previously described^29^. The total time spent in the dark and light compartments, and the delay to cross from the dark to the light chamber for the first time were used as parameters for analysis. The three-chambered social test (3CST) was performed as previously described^42^ in a rectangular apparatus (60 cm long, 40 cm wide and 22 cm deep; Ugo Basile). Two grid enclosures were placed in opposite chambers (left or right). During the habituation session, the experimental mouse was placed into the central chamber and allowed to explore freely for 10 min. Then, the test mouse was contained in the central chamber for 1 minute while the experimenter placed a new age-matched wild-type stranger mouse (stranger) in one of the two grid enclosures (the sides were alternated between trials to cancel side bias). The sociability test (ST) session was evaluated during a 10-minute trial from the moment when the passageways were opened, and the test mouse allowed to access all three chambers. We scored ST using two individual measures: (1) time in the chamber; and (2) time in the contact zones (a 3.5 cm zone surrounding the grid enclosure).

For all behavior tests, mice were acclimatized to a dedicated behavioral room for 1 h before testing and the experimenter was blind to the experimental group of the animal while running the experiment. The video-camera was mounted above the apparatus and behavior measurements were scored using an automated video-tracking software (ANYmaze). All behavior tests were performed 21 days after the viral injections.

### Statistical analysis

Normality was tested using the D’Agostino-Pearson omnibus test (alpha < 0.05) and homogeneity of variance with Brown-Forsythe’s test (alpha < 0.05). Post-hoc testing was performed using Tukey’s or Sidak’s multiple comparisons tests for two-way ANOVA. One-way ANOVA followed by Bonferroni’s multiple comparison and unpaired Student’s *t*-tests were performed to compare three or two groups with a single variable, respectively. The experimenter was blinded to the experimental groups while running the statistical analysis. All tests were two-tailed and had an alpha level of 0.05. All statistical analysis was performed using GraphPad Prism version 6 (GraphPad Software).

## Results

### Valproate induces Ncs-1 expression

**Figure 1:**
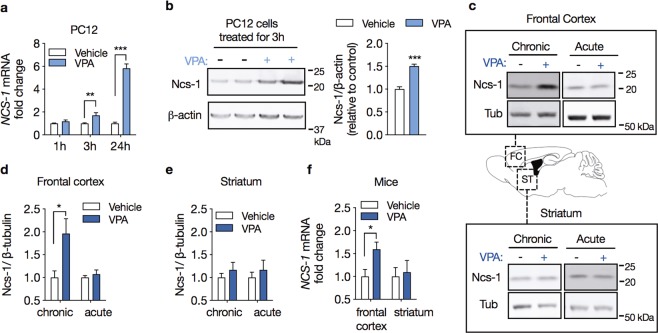
Valproate induces Ncs-1 expression. (**a**) Temporal pattern for *Ncs-1* mRNA expression in response to 0.62 mM VPA treatment relative to vehicle-treated PC12 cells (n = 3/group). (**b**) Western blot analysis (left) and densitometry quantification (right) of Ncs-1 protein expression in PC12 cells treated with 0.62 mM VPA or vehicle for 3 h (n = 4/group). **(c)** Representative western blot analysis of Ncs-1 protein expression in frontal cortex (upper panel) or striatum (lower panel) of mice chronically (25 g of drug per 1 kg of chow for 21 days) or acutely (400 mg/kg i.p. for 3 h) administered with VPA (+) or vehicle (−). The bands of interest were cropped from the same membrane. **(d**,**e)** Summary of Ncs-1 protein expression in mouse frontal cortex (**d**) and striatum (**e**) after VPA or vehicle treatment (n = 5/group). **(f)** *Ncs-1* mRNA expression following chronic VPA treatment in mouse frontal cortex and striatum relative to vehicle-treated animals (n = 5/group). In all cases data are represented as mean ± SEM. Western blot quantifications were normalized against β-actin or β-tubulin and plotted relative to vehicle-treated samples, which was set to 1. Significant differences were determined by two-way ANOVA followed by Sidak’s multiple comparison tests (**a**) or Student’s t-tests (**b**–**f**). Asterisks (*) in the figures indicate the p-values for the post-hoc test and correspond to the following values: *p < 0.05; **p < 0.01; ***p < 0.001, based upon mean ± standard error of mean.

We investigated the time course of changes in *Ncs-1* gene expression in response to 0.62 mM VPA in PC12 cells. This concentration was used based on the levels of VPA in the serum from treated patients^43^. We found that *in vitro* VPA treatment progressively increased Ncs-1 mRNA levels, with initial effects at 3 h (1.7-fold increase) and peak levels after 24 h of treatment, which showed a 5.8-fold increase (interaction effect_treatment × time_: F_2,12_ = 85.05, p < 0.001; n = 3/group, two-way ANOVA; Fig. 1a). This VPA treatment also increased the Ncs-1 protein expression (50%) in PC12 cells treated for 3 h (VPA vs. vehicle: mean difference (MD) = 0.50, 95% CI [0.34 to 0.67], t_(6)_ = 7.48, p < 0.001, n = 4/group, Student’s t-test; Fig. 1b). As Ncs-1 upregulation has been observed in injured neurons^3^, we determined if VPA caused cell damage, which itself might explain the upregulation of Ncs-1 expression. PC12 cells were incubated with a concentration range of VPA (0.62–5 mM) for 24 h, however MTT and Hoechst 33342 labeling assays did not show cytotoxicity at either VPA concentrations (VPA mean survival: 0.62 mM: 100.2 ± 3.34; 1.25 mM: 109.6 ± 8.21; 2.5 mM: 126.3 ± 6.69; 5.0 mM: 117.5 ± 8.30; F_3,12_ = 2.50, p = 0.109; n = 4/group, one-way ANOVA; Supplementary Fig. S1).

To establish whether VPA increases Ncs-1 expression *in vivo*, we analyzed tissue from mice chronically or acutely treated with VPA. Ncs-1 measurements were performed in the frontal cortex and striatum, two brain regions implicated in neuropsychiatric disorders and responsiveness to mood-stabilizing drugs^44,45^. We found a significant upregulation of both Ncs-1 protein (96%) and mRNA (1.6-fold increase) that were restricted to the frontal cortex of mice chronically treated with VPA (VPA vs. vehicle (protein): MD = 0.96, 95% CI [0.14 to 1.78], t_(6)_ = 2.717, p = 0.026; VPA vs. vehicle (mRNA): MD = 0.59, 95% CI [0.09 to 1.09], t_(6)_ = 2.718, p = 0.026; n = 5/group, Student’s t-tests; Fig. 1c–f). Notably, these VPA effects were not observed following acute VPA administration (Fig. 1c–f).

### Selective inhibitors of HDAC do not increase Ncs-1 expression

**Figure 2:**
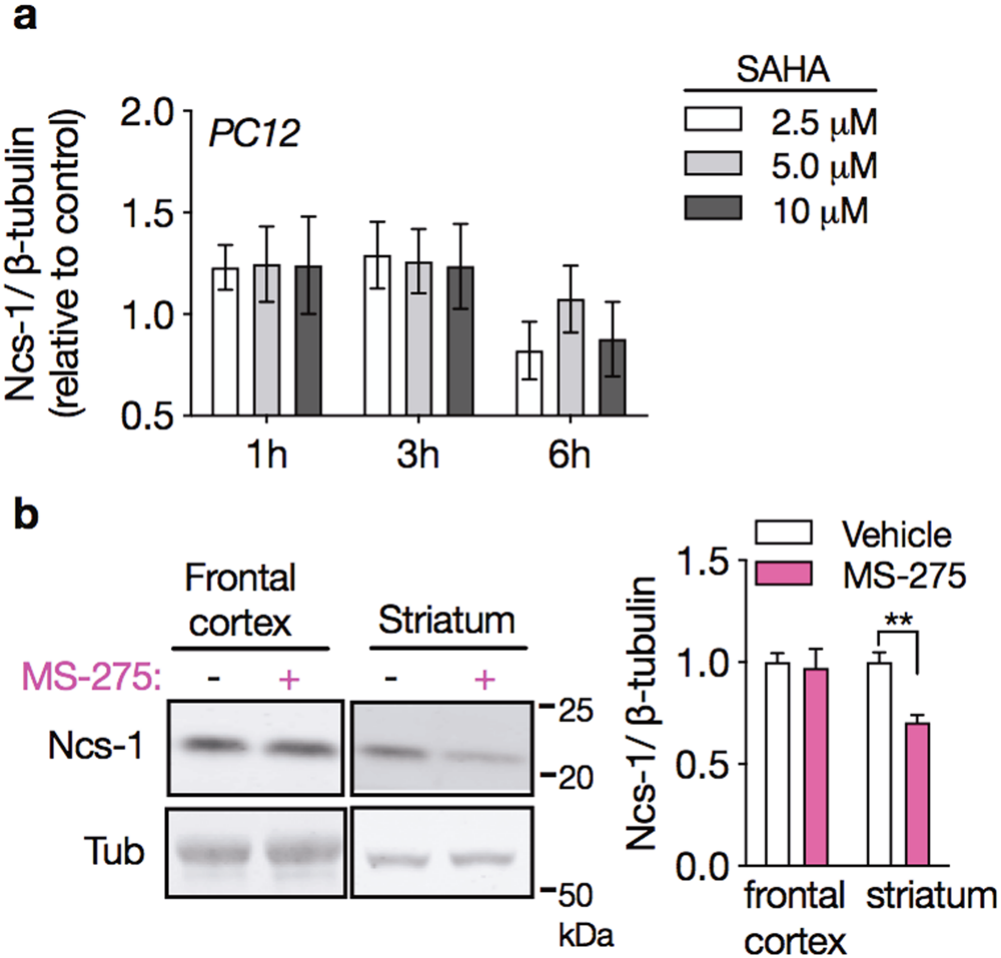
HDAC inhibitors do not induce Ncs-1 expression. **(a)** Time course and dose response of Ncs-1 protein expression in SAHA-treated PC12 cells (n = 4/group). **(b)** Representative western blot (left) and densitometry quantification (right) of Ncs-1 protein expression in frontal cortex or striatum of mice chronically injected with MS-275 (20 mg/kg i.p. daily for 21 days) or vehicle (n = 5/group). In all cases data are represented as mean ± SEM. The bands of interest were cropped from the same membrane. Western blot quantifications were normalized against β-tubulin and plotted relative to vehicle-treated samples, which was set to 1. Significant differences were determined by two-way ANOVA followed by Tukey’s multiple comparison tests (**a**) or Student’s t-tests (**b**). Asterisks (*) in the figures indicate the p-values for the post-hoc test and correspond to the following values: *p < 0.05; **p < 0.01; ***p < 0.001, based upon mean ± standard error of mean.

Treatment with VPA has been linked to changes in gene expression caused by inhibition of the catalytic activity of class I histone deacetylase (HDAC1)^20,46^. To investigate whether HDAC inhibition upregulates Ncs-1 expression, we first treated PC12 cells with the broad-spectrum HDAC inhibitor SAHA^47^. PC12 cells treated with three different concentration of SAHA for up to 6 h displayed similar Ncs-1 protein expression when compared with vehicle-treated cells (interaction effect_treatment × time_: F_6,36_ = 0.52, p = 0.786; n = 4/group, two-way ANOVA; Fig. 2a). We also injected mice with the HDAC1 inhibitor MS-275 for 21 days to examine if the lack of effect of HDAC inhibition on Ncs-1 expression is replicated *in vivo*^48^. In agreement with results from cells, chronic MS-275 treatment had no detectable effect on Ncs-1 protein expression in mice frontal cortex (frontal cortex: MD = 0.02, t_(8)_ = 0.273, p = 0.972; striatum: MD = 0.29, t_(8)_ = 4.961, p = 0.001; n = 5/group, Student’s t-tests; Fig. 2b). Conversely, MS-275 administration decreased Ncs-1 protein expression in the striatum (Fig. 2b). Thus, these findings do not support HDAC-mediated epigenetic mechanisms in the regulation of Ncs-1 by VPA.

### Inhibition of Gsk3β is sufficient to upregulate Ncs-1 expression

**Figure 3:**
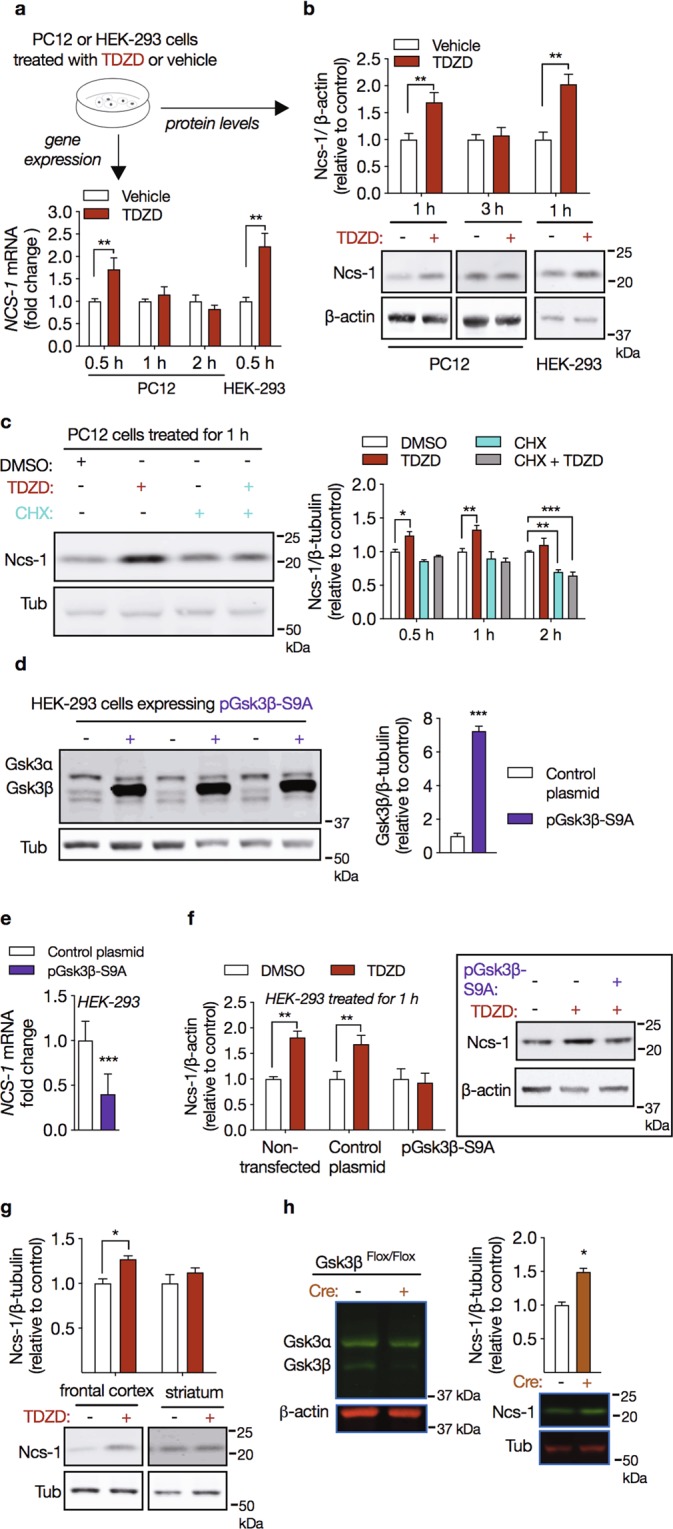
Gsk3β regulates Ncs-1 expression. (**a**) Temporal pattern for *Ncs-1* gene expression in PC12 or HEK-293 cells treated with the Gsk3 inhibitor TDZD-8 (10 µM) relative to experimental control (DMSO) (n = 4/group). **(b)** Western blot analysis of Ncs-1 protein expression in PC12 (1 h and 3 h) or HEK-293 cells (1 h) treated with TDZD-8 (10 µM, +) relative to experimental control (DMSO, −) (n = 4/group). The bottom panel shows the representative western blots cropped from the same membrane obtained from these experiments. **(c**) Representative western blots cropped from the same membrane (left) and densitometry analysis (right) showed that 30 min pretreatment with protein synthesis inhibitor cycloheximide (CHX, 10 μg/ml) blocked TDZD-8-induced Ncs-1 expression (n = 4/group). **(d)** Representative western blots cropped from the same membrane (left) and densitometry analysis (right) of HEK-293 cells transiently transfected with a plasmid encoding the constitutively active Gsk3β mutant (pGsk3β-S9A, +) or a cloning vector alone (control plasmid, −) (n = 6/group). **(e)** Overexpression of Gsk3β-S9A decreased *NCS-1* mRNA levels (n = 6/group). **(f)** Overexpression of Gsk3β-S9A also abolished TDZD-8-induced Ncs-1 protein expression (n = 6/group). Control plasmid expressed GFP in the same plasmid backbone used for GSK3beta-S9A expression. The panel in the right shows a representative western blot cropped from the same membrane obtained from this experiment. **(g)** Ncs-1 protein expression in frontal cortex and striatum of mice administered with TDZD-8 (30 mg/kg i.p. for 1 h, +) or vehicle (−) (n = 5/group). The bottom panel displays representative western blots cropped from the same membrane from these experiments. (**h**) Representative western blot cropped from the same membrane of Gsk3 isoforms in frontal cortex of Cre-positive (+) or Cre-negative (−) CamKII-Gsk3β^Flox/Flox^ mice. Downregulation of Gsk3β increases Ncs-1 protein expression in the frontal cortex of Cre-positive CamKII-Gsk3β^Flox/Flox^ mice (n = 5/group). The bottom panel shows a representative western blot cropped from the same membrane obtained from this experiment. In all cases data are represented as mean ± SEM. Western blot quantifications were normalized against β-actin (or β-tubulin) and plotted relative to vehicle-treated samples, which was set to 1. Significant differences were determined by Student’s t-tests (HEK-293 data in **a**,**b**, **d**–**h**) or two-way ANOVA followed by Sidak’s multiple comparison tests (PC12 data in **a**,**b**, and **c**). Asterisks (*) in the figures indicate the p-values for the post-hoc test and correspond to the following values: *p < 0.05; **p < 0.01; ***p < 0.001, based upon mean ± standard error of mean. The drawing in (**a**) was created with the Keynote software version 9.2.1 (http://apple.com/keynote).

Chronic VPA administration has been shown to inhibit the activity of Gsk3β in mouse brain^29^. To test the possibility that inhibition of Gsk3 increases Ncs-1 expression, we treated PC12 and HEK-293 cells with the selective Gsk3 inhibitor TDZD-8^49^. Both PC12 and HEK-293 cells displayed increases in Ncs-1 mRNA upon 10 μM TDZD-8 treatment. TDZD-8 treatment caused a 1.7-fold increase in Ncs-1 mRNA levels in PC12 cells that did not last for more than 30 minutes (PC12: interaction effect_treatment × time_: F_2,18_ = 4.73, p = 0.022; vehicle vs. VPA at 0.5 h: p = 0.008; n = 4/group, two-way ANOVA; HEK-293 at 0.5 h: MD = 1.22, t_(6)_ = 4.086, p = 0.006, n = 4/group, Student’s t-tests; Fig. 3a). Western blot analyses showed that TDZD-8 also induced Ncs-1 protein expression in both PC12 and HEK-293 cells that was observed after 1 h of treatment. No further increase in Ncs-1 protein expression occurred when PC12 cells were treated for up to 3 h (PC12: interaction effect_treatment × time_: F_1,12_ = 4.935, p = 0.046; vehicle vs. TDZD-8 at 1 h: p = 0.008; n = 4/group, two-way ANOVA; HEK-293 at 1 h: MD = 1.02, t_(6)_ = 4.416, p = 0.004, n = 4/group, Student’s t-tests; Fig. 3b).

To investigate whether upregulation of Ncs-1 following TDZD-8 treatment results from newly synthesized Ncs-1 protein, PC12 cells were pretreated with the protein synthesis inhibitor cycloheximide (CHX) and 30 min later TDZD-8 or vehicle were added to the cultured cells for additional 30, 60 or 120 min. Western blot analysis showed that exposure to CHX prevented the upregulation of Ncs-1 by TDZD-8, indicating that increased Ncs-1 protein expression in response to Gsk3 inhibition involves *de novo* protein synthesis (interaction effect_treatment × time_: F_6,36_ = 1.614, p = 0.171; main effect_treatment_: F_3,36_ = 36.43, p < 0.001; main effect_time_: F_2,36_ = 10.0, p < 0.001; DMSO vs. TDZD-8 at 1 h: MD = 0.23, p = 0.001; DMSO vs. CHX at 1 h: MD = 0.14, p = 0.282; DMSO vs. CHX + TDZD-8 at 1 h: MD = 0.06, p = 0.827; n = 4/group, two-way ANOVA; Fig. 3c).

To further establish that Gsk3β inhibits *NCS-1* gene expression, we transfected HEK-293 cells with a plasmid encoding a constitutively active Gsk3β mutant, in which the N-terminal inhibitory Ser9 residue has been mutated to an alanine (pGsk3β-S9A). HEK-293 cells transfected with pGsk3β-S9A (HEK-293-Gsk3β-S9A) displayed a 7-fold increase in Gsk3β protein levels (MD = 6.247, t_(10)_ = 18.33, p < 0.001, n = 6/group, Student’s t-tests; Fig. 3d). In line with data obtained using TDZD-8, HEK-293-Gsk3β-S9A cells displayed a 60% reduction in *NCS-1* mRNA levels (MD = −0.59, t_(10)_ = 4.70, p < 0.001; n = 6/group, Student’s t-tests; Fig. 3e). Moreover, overexpression of Gsk3β-S9A prevented the induction of Ncs-1 expression by TDZD-8 (DMSO vs. TDZD-8 [Non-transfected]: MD = 0.81, p < 0.001; DMSO vs. CHX [Control plasmid]: MD = 0.68, p = 0.014; DMSO vs. TDZD-8 [pGsk3β-S9A]: MD = 0.07, p = 0.796; n = 6/group, Student’s t-tests; Fig. 3f).

We next used pharmacological and genetic approaches to investigate the effect of Gsk3 inhibition on Ncs-1 expression *in vivo*. First, mice were injected with a single dose of TDZD-8 (30 mg/kg i.p. for 1 h) and western blot analysis indicated that *in vivo* Gsk3 inhibition produced a significant increase in Ncs-1 protein expression in the frontal cortex (MD = 0.27, t_(8)_ = 4.27, p = 0.002, n = 5/group, Student’s t-tests; Fig. 3g). Conversely, TDZD-8 caused no change in Ncs-1 protein expression in striatum (MD = 0.12, t_(8)_ = 1.092, p = 0.306, n = 5/group, Student’s t-tests; Fig. 3g). Second, the expression pattern of Ncs-1 in conditional Gsk3β knockout mice^38^ was examined. These mice carry floxed Gsk3β alleles, which are inactivated after birth by a CamKII promoter driven Cre recombinase only in forebrain pyramidal neurons. We observed that Ncs-1 protein expression was increased in the frontal cortex of Cre-positive in comparison to Cre-negative CamKII-Gsk3β^Flox/Flox^ mice (MD = 0.49, t_(6)_ = 6.853, p = 0.705, n = 5/group, Student’s t-tests; Fig. 3h).

Inhibition of Gsk3 by chronic VPA treatment has been reported to require D2R and βArr2 expression^29,39^. D2R and βArr2 KO mice displayed equivalent levels of Ncs-1 protein expression in frontal cortex when compared to WT littermates (Supplementary Fig. S2a). However, chronic VPA treatment in D2R-KO or βArr2-KO mice failed to induced Ncs-1 upregulation in the frontal cortex as compared to their respective WT littermates (βArr2 WT: MD = 0.81, t_(8)_ = 4.343, p = 0.002; βArr2 KO: MD = 0.11, t_(8)_ = 0.465, p = 0.653; D2R WT: MD = 0.69, t_(8)_ = 4.181, p = 0.003; D2R KO: MD = −0.18, t_(8)_ = 0.462, p = 0.656, n = 5/group, Student’s t-tests; Supplementary Fig. S2b,c). This suggests that Ncs-1 upregulation by VPA may depend on VPA-mediated inhibition of Gsk3β through the D2R/βArr2 signaling cascade.

### Overexpression of the RNA binding protein Fxr1 do not change Ncs-1 expression

Fxr1 is an RNA binding protein that regulates posttranscriptional steps of gene expression^50^. Chronic VPA increases Fxr1 levels through inhibition of Gsk3β, and this mechanism contributes to evoke behavioral responses in mice^29,35^. Since the upregulation of Ncs-1 by VPA requires protein synthesis (Fig. 3c), we hypothesized that Gsk3β inhibition may induce Ncs-1 protein expression by increasing its translation through recruitment of Fxr1. We overexpressed Fxr1 in either N2A cells or mice mPFC, however expression levels of Ncs-1 protein were similar in these conditions. These findings indicate that Ncs-1 expression is not regulated by Fxr1 and suggest that Gsk3 regulate Ncs-1 via a different mechanism.

### Activity of the human NCS-1 gene promoter

**Figure 4:**
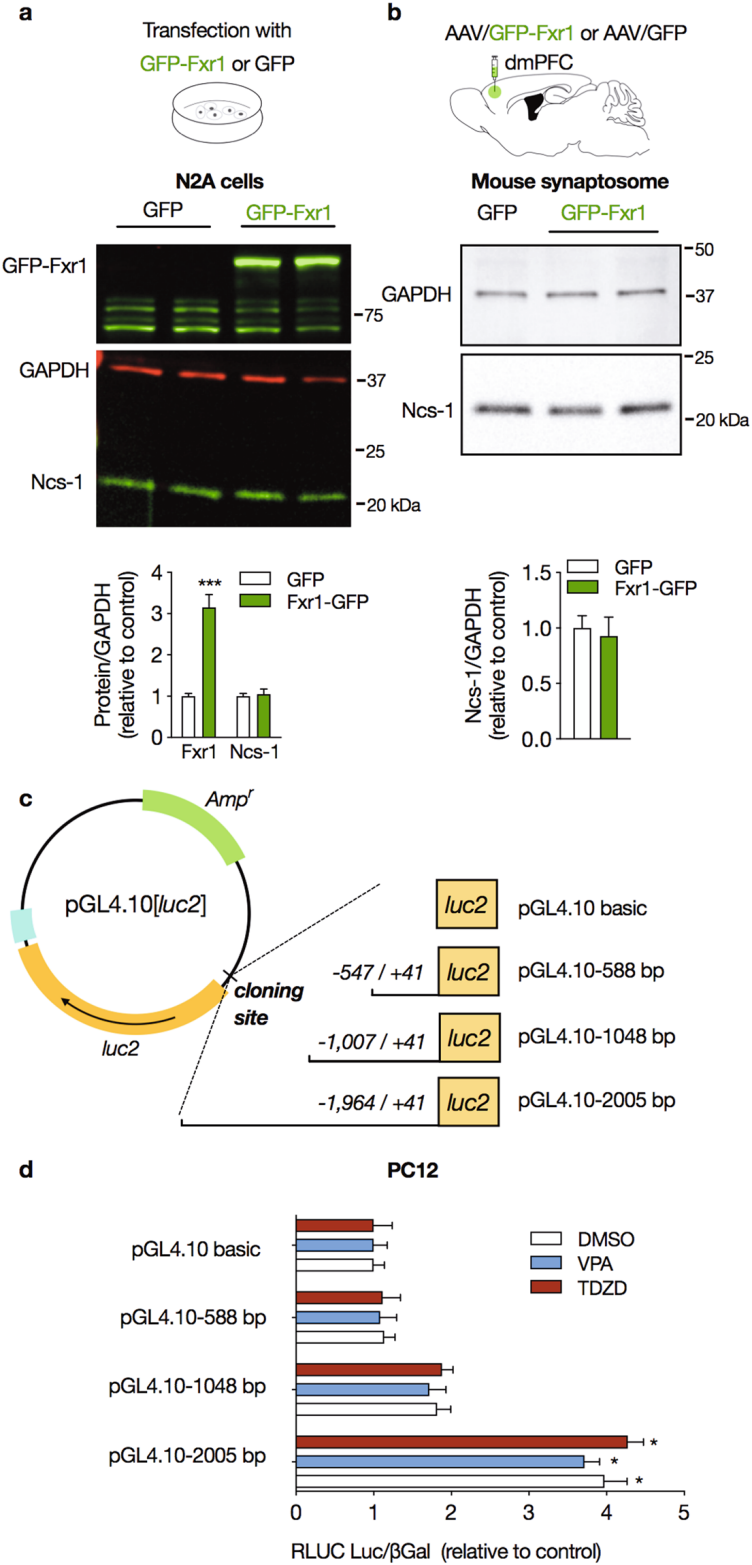
Effect of Fxr1 on Ncs-1 expression, and activity of *NCS-1* gene promoter. (**a**) Representative western blot (upper panel) and densitometry analysis (lower panel) of Fxr1 and Ncs-1 protein expression following infection with either GFP-Fxr1 or GFP plasmids in N2A cells (n = 4–5/group). (**b**) Representative western blot cropped from the same membrane (upper panel) and densitometry analysis (lower panel) of Ncs-1 protein expression in purified synaptosome from dorsomedial prefrontal cortex (dmPFC) of mice injected with either AAV/GFP-Fxr1 or AAV/GFP (n = 4–5/group). **(c)** Schematic representation of the three Ncs-1 reporter constructs (pGL4.10–588, pGL4.10–1048 and pGL4.10–2005) cloned into the pGL4.10[*luc2*] promoterless vector containing a luciferase (*luc2*) reporter gene. (**d)** Luciferase activity was measured in PC12 cells transfected and treated with DMSO (0.001% for 1 or 3 h), VPA (0.625 mM for 3 h) or TDZD-8 (10 µM for 1 h). Relative levels of luciferase activity (RLUC) were normalized by the pGL4.10 cloned with inserts of similar lengths isolated from exon 5 of the *CKAMP44* gene, and co-transfection with the pCMV-βgal vector encoding β-galactosidase (see the Supplementary Table S1 for information about the reporter constructs). Luciferase activity was detected only in PC12 cells transfected with pGL4.10–2005. The bars represent the mean ± SEM values of relative luciferase activity from at least four different transfections carried out in triplicate. Significant differences were determined by Student’s t-tests (**a,b**) or two-way ANOVA followed by Tukey’s multiple comparison tests (**c,d**). Asterisks (*) in the figures indicate the p-values for the post-hoc test and correspond to the following values: *p < 0.05; **p < 0.01; ***p < 0.001, based upon mean ± standard error of mean. The drawings in (**a–c**) were created with the Keynote software version 9.2.1 (http://apple.com/keynote).

Several targets of Gsk3β signaling are transcription factors, such as Creb and β-catenin^24,51,52^. We thus developed a reporter assay system to investigate the action of VPA and Gsk3 activity on Ncs-1 gene transcription. A 2,005 bp nucleotide sequence from the predicted human *NCS-1* promoter sequence (from *NCS-1* TSS +41 to −1,964) was cloned upstream of the firefly luciferase gene in the pGL4.10[luc2] basic vector (Fig. 4c). TATA boxes, higher concentration of GpC islands and numerous DNA binding domains for transcription factors (including c-Ets, Creb and AP1) were predicted in this sequence (Fig. S3). PC12 cells transfected with pGL4.10–2005 displayed 4-fold more normalized luciferase activity (RLU) than PC12 transfected with pGL4.10[luc2] (Fig. 4d). In contrast, no significant increase in RLU was detected in PC12 cells transfected with the two reporter constructs carrying deletions (Fig. 4d), thus indicating the whole +41 to −1,964 sequence is necessary for promoter activity. Treatment with VPA or TDZD-8 did not change RLU of transfected PC12 cells (Fig. 4d).

### Ncs-1 overexpression the frontal cortex induces behavioral effects

**Figure 5:**
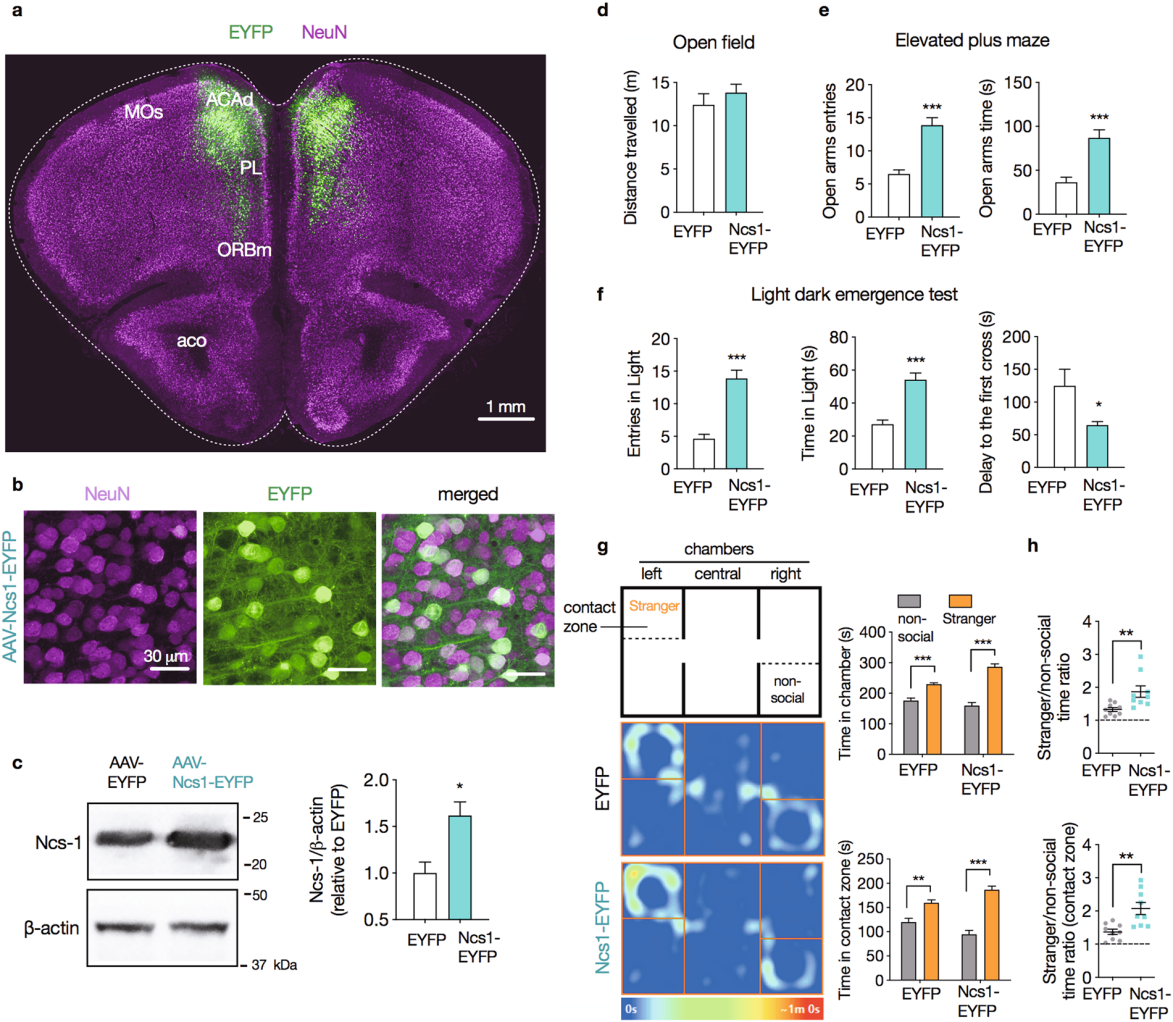
Overexpression of Ncs-1 in frontal cortex reduces anxiety-like and increases social behavior in mice. (**a**) Mice received bilateral stereotaxic injections of viral particles containing either AAV/hSyn-NCS1-EYFP (Ncs1-EYFP mice) or AAV/hSyn-EYFP (EYFP mice) into dorsomedial prefrontal cortex (dmPFC). Green, eYFP from AAV; magenta, NeuN from immunoreactivity. MOs: secondary motor area; ACAd: anterior cingulate area, dorsal part; PL: prelimbic area; ORBm: orbital area, medial part; aco: anterior commissure, olfactory limb. **(b**) High magnification sections displaying Ncs1-EYFP expression in both cell body and projections. These cells co-stained with the neuronal marker NeuN at the injection site. (**c)** Representative western blot cropped from the same membrane and densitometry analysis of Ncs-1 protein expression in frontal cortex of Ncs1-EYFP and EYFP mice (n = 5–6/group). (**d**–**h**) Mice were subjected to behavioral evaluation 3 weeks following AAV injections (n = 8–9/group). Behavior was scored in the open field (**d**), elevated plus maze (**e**), dark light emergence test (**f**), and (**g**,**h**) three-chamber sociability test. Sociability tests are presented as the time spent in each chamber or contact zone, and time ratio. Heat-map plots show the representative maximum occupancy across all the tests in the entire experiment. Data are means ± SEM. Significant differences were determined by Student t-tests (**c–f,h**) or two-way ANOVA followed by Sidak’s multiple comparison tests (**g**). Asterisks (*) in the figures indicate the p-values for the post-hoc test and correspond to the following values: *p < 0.05; **p < 0.01; ***p < 0.001, based upon mean ± standard error of mean.

To establish the behavioral effects of Ncs-1 upregulation in response to VPA, mice received bilateral stereotaxic injections of viral particles containing either AAV/hSyn-NCS1-EYFP (Ncs1-EYFP mice) or AAV/hSyn-EYFP (EYFP mice) into dmPFC (Fig. 5a). High magnification confocal imaging (100×) conducted four weeks after viral injection, confirmed Ncs1-EYFP expression in a large subset of neuronal bodies and processes (Fig. 5b), corresponding, on average, 65% of the dmPFC NeuN+ cells. These injections produced a 60% increase in total Ncs-1 levels in frontal cortex of Ncs1-EYFP mice compared with EYFP mice (MD = 0.59, t_(9)_ = 3.226, p = 0.010, n = 5–6/group, Student’s t-tests; Fig. 5c).

Mice were subjected to behavioral tests^29^ three weeks following viral injections. These tests included elevated plus maze (EPM), dark light emergence test (DLET) and open field (OFT) to evaluate effects on anxiety related behaviors. A 3-chambered sociability tests (3CST) was used to evaluate the effects of Ncs-1 on social behavior. These tests were selected on the base of reported shared behavioral effects of VPA^29^ and Gsk3 inhibition^35,38^ in mice.

Collectively, these evaluations revealed that bilateral injection of AAV/hSyn-Ncs1-EYFP produced anxiolytic-like and pro-social behaviors (Fig. 5d–h). Our data showed that Ncs1-EYFP mice made more entries and spent more time in the open arms of the EPM (open arms entries: MD = 7.37, t_(14)_ = 5.727, p < 0.001; open arms time [s]: MD = 50.71, t_(14)_ = 4.757, p < 0.001; n = 8/group, Student’s t-tests; Fig. 5e). Ncs1-EYFP mice also made more entries and spent more time in the light compartment in the DLET, while displaying a lower delay to visit the light chamber for the first time (entries in light: MD = 9.25, t_(14)_ = 6.406, p < 0.001; time in light [s]: MD = 27.00, t_(14)_ = 5.532, p < 0.001; delay to the first cross: MD = −60.38, t_(14)_ = 2.345, p < 0.001; n = 8/group, Student’s t-tests; Fig. 5F). These behaviors were not affected by changes in locomotor behavior as the experimental groups shared similar distance travelled in OFT (MD = 1.38, t_(14)_ = 0.857, p = 0.405; n = 8/group, Student’s t-tests; Fig. 5d).

During the sociability test (ST), both Ncs1-EYFP and EYFP mice spent more time in the chamber with the social stimulus (stranger 1) than the chamber with the non-social stimulus (grid enclosure only) (interaction effect_chamber × AAV_: F_1,32_ = 18.71, p < 0.001; n = 8/group, two-way ANOVA; Fig. 5g). However, time ratio between social and non-social chambers was greater in Ncs1-EYFP (1.87 ± 0.17 in Ncs1-EYFP vs. 1.33 ± 0.06 in EYFP mice, t_(16)_ = 2.951, p = 0.009; n = 8/group, Student’s t-tests; Fig. 5h). We observed similar results when we analyzed data from the contact zone instead (interaction effect_chamber × AAV_: F_1,32_ = 12.35, p = 0.001, n = 9/group, two-way ANOVA; Fig. 5g,h). We found no differences in side bias between the experimental groups, as shown by similar time spent in the left and right chambers during the habituation session (time spent in left vs. right chamber: EYFP = 224.91 ± 14.47 vs. 214.88 ± 13.47 s; Ncs1-EYFP = 219.18 ± 15.53 vs. 207.48 ± 14.55 s; p = 0.081, n = 9/group, two-way ANOVA).

## Discussion

Decreases in Ncs-1 expression may contribute to the emergence of behavioral abnormalities. For example, bipolar disorder patients present decreased expression of Ncs-1 in leukocytes^6^, and Ncs-1 deficiency in mice triggers depressive-like, anxiety-like and impaired motivated behaviors^10,11^. We found that chronic treatment with the mood stabilizer VPA upregulates Ncs-1 in cell lines and the mouse frontal cortex (Fig. 1), which is likely to be mediated by inhibition of Gsk3 (Figs. 2, 3 and S2). In addition, overexpression of Ncs-1 in the frontal cortex of mice was accompanied by anxiolytic-like and increased social behavior (Fig. 5), suggesting a role of Ncs-1 in mediating effects of VPA and Gsk3 inhibition.

Previous studies showed that VPA can increase gene expression through the inhibition of histone deacetylases (HDACs)^22,53,54^, however it is unlikely that VPA mediates Ncs-1 upregulation through this pathway since both *in vitro* and *in vivo* treatments with HDAC inhibitors did not induce Ncs-1 expression (Fig. 2). On the other hand, both pharmacological inhibition and genetic downregulation of Gsk3β increased Ncs-1 expression, which was restricted to the frontal cortex in mice (Fig. 3). In contrast to the chronic treatment, it is possible that the acute VPA treatment (400 mg/kg) did not upregulate Ncs-1 in the frontal cortex due to its inability to inhibit Gsk3^29^. Therefore, VPA-mediated Ncs-1 upregulation seems to be related to Gsk3 inhibition.

Chronic VPA administration has been reported to inhibit Gsk3 in both the frontal cortex and striatum^29^. However, our findings showed that Gsk3 inhibition in the striatum is not able to induce Ncs-1 protein expression. These contrasting effects strength the evidence that Gsk3 inhibition evoke distinct molecular changes across brain regions and cell types^24^. Upregulation of Ncs-1 protein level in response to Gsk3 inhibition is dependent upon *de-novo* protein synthesis (Fig. 3c). This suggests that Gsk3 may affect mRNA translation and/or the transcription of the *Ncs-1* gene. Interestingly, inhibition of Gsk3 in response to VPA has previously been shown to result in a degradation of Fxr1 that is also associated with the behavioral effect of VPA in mice^29^. Since Fxr1 can regulate mRNA translation, we thus verified its effect on Ncs-1 protein levels and found no impact of Fxr1 overexpression on Ncs-1 protein levels (Fig. 4) thus suggesting that upregulation of Fxr1 and Ncs-1 are two independent effects of Gsk3 inhibition and VPA treatment which may both affect behavioral responses independently.

The transcriptional regulation of the human *NCS-1* gene remains completely unexplored. We found that Gsk3 inhibition increases *NCS-1* mRNA levels (Fig. 3). Based on the effects of TDZD-8 on *NCS-1* mRNA levels (30 min) (Fig. 3a), we hypothesized that upregulation of Ncs-1 may reflect an effect of Gsk3 on *Ncs-1* gene expression. We thus conducted a preliminary characterization of the *NCS-1* core gene promoter, which has not been previously described. Our reporter assay using a 2,005 bp upstream sequence of the predicted human *NCS-1* promoter displayed a 4-fold increase in luciferase activity when the reporter construct was expressed in PC12 cells (Fig. 4d). However, our observations also indicate that the *NCS-1* core promoter activity is not affected by Gsk3 inhibition. Thus, while the effect of VPA and Gsk3 inhibition on the expression of Ncs-1 mRNA and protein most probably involved increased *Ncs-1* gene transcription, our results indicate that this action may involve a larger promoter sequence or a more complex pattern of promoter/enhancer regulation.

Viral-vector-driven Ncs-1 overexpression in the dorsomedial prefrontal cortex (dmPFC) was sufficient to promote anxiolytic-like and social behavior (Fig. 5). These effects were similar to those reported previously in response to chronic lithium and VPA^29^ or of local cortical Gsk3β inactivation^35,38^. These observations are also consistent with previous studies showing that Ncs-1 deficiency increases depression/anxiety-related behaviors^10^ and impaired motivated behaviors in mice^11^.

One possible mechanism for these behavioral effects is that Ncs-1 upregulation may impact on dopamine (DA) signaling and receptor desensitization to regulate behavior. Ncs-1 has been shown to inhibit D2R-βArr2 mediated internalization, and strengthens G-protein-mediated D2R signaling^2^. Increased levels of Ncs-1 are thus likely to increase the post-synaptic G-protein mediated DA response and to alter the balance between G-protein mediated and βArr2 mediated signaling downstream of this receptor^32,55^. Interestingly D2R expression has been reported in several populations of cortical neurons, including principal neurons and multiple interneuron subtypes of the mouse prefrontal cortex^56^. However, the functions of neuronal Ncs-1 are still mostly unexplored and it is probable that other mechanisms can also be involved in the regulation of cortical neurons and associated behavior. Study of Ncs-1 function specifically in different subtypes of cortical neurons will thus be needed to fully identify the mechanisms by which Ncs-1 contributes to the regulation of social and mood-associated behaviors.

In conclusion, our findings uncovered an effect of chronic VPA treatment on Ncs-1 expression that is mediated through inhibition of Gsk3. These data support a potential contribution of Ncs-1 to the behavioral effects of mood stabilizers and provide a mechanism by which inhibition of Gsk3 affects mood-related behavioral response.

## Supplementary information

Supplementary Information.

## Data Availability

All the data that support the findings presented in this study are available from the corresponding author upon request.
