# Supplementary material for: Contribution of neuronal calcium sensor 1 (Ncs-1) to anxiolytic-like and social behavior mediated by valproate and Gsk3 inhibition
Source: Sci Rep. 2020 Mar 12;10:4566. doi: 10.1038/s41598-020-61248-z (PMC7067888; doi:10.1038/s41598-020-61248-z)

Supplementary Material

Contribution of Neuronal calcium sensor 1 (Ncs-1) to anxiolytic-like and social behavior mediated by valproate and Gsk3 inhibition

Luiz Alexandre Viana **Magno** PhD[1,2,3], Helia **Tenza-Ferrer** PhD [1,2], Mélcar **Collodetti**[1,2], Eduardo de Souza **Nicolau** MSc[1,2], Jivan **Khlghatyan** PhD [3,4], Thomas **Del'Guidice** PhD [3,5], Marco Aurélio **Romano-Silva** PhD [1,2]* and Jean Martin **Beaulieu** PhD [3,4]*

Table S1. Gene position and primers used to amplify *NCS-1* and *CKAMP44* inserts cloned into pGL4.10[luc2] promoterless vector.

Gene/ Length (bp)	Location (GRCh38.p12)	Forward primer (5' - 3')	Reverse primer (5' - 3')
NCS-1			
2,005	9: 130,170,614–130,172,618	AGATGAGGTCTCCCCTGTCA	GGCGCCCAGCAGGAG
1,048	9: 130,171,571–130172618	ATTCAGGTGGAGCCTGTCAC	GGCGCCCAGCAGGAG
588	9: 130,172,031–130,172,618	CCGTGTCGAATGAATGAATG	GGCGCCCAGCAGGAG
CKAMP44[b]			
2,063	16: 13,235,214 – 13,237,276	GGCCTACACCTCTACCACCA	CAACTGCATTGTTGGTGTCC
1,017	16: 13,235,214 – 13,236,230	GGCCTACACCTCTACCACCA	ATACTCTCCGGACCCTCTCC
614	16: 13,235,214 – 13,235,827	GGCCTACACCTCTACCACCA	CAGCACGTGGCAAGTAGTGT

[a] Gene position is displayed as a relative base position downstream (+) or upstream (-) of transcriptional start site of *NCS-1* gene. [b] *Cystine-knot AMPAR modulating protein*.

Table S2. Primers used for relative quantification of mRNA.

Gene	Specie	Forward primer (5' - 3')	Reverse primer (5' - 3')
Ncs-1	Rat	GCAGTGGTACAAGGGTTTCA	CCGATAGAGCCTGGATGAAT
	Mouse	AAGCCTCGGACTTTGAGACA	CTGGACCACACCCCTAAAGA
	Human	GCCGCCGAGGATGGGGAAATC	AGCTTGAAGGCCCACCGTAGC
Beta-actin	Mouse/Rat	AGCCATGTACGTAGCCATCC	CTCTCAGCTGTGGTGGTGAA
	Mouse/Rat	GCACAGAGCCTCGCCTTTGCC	CATGCCCACCATCACGCCCTGG

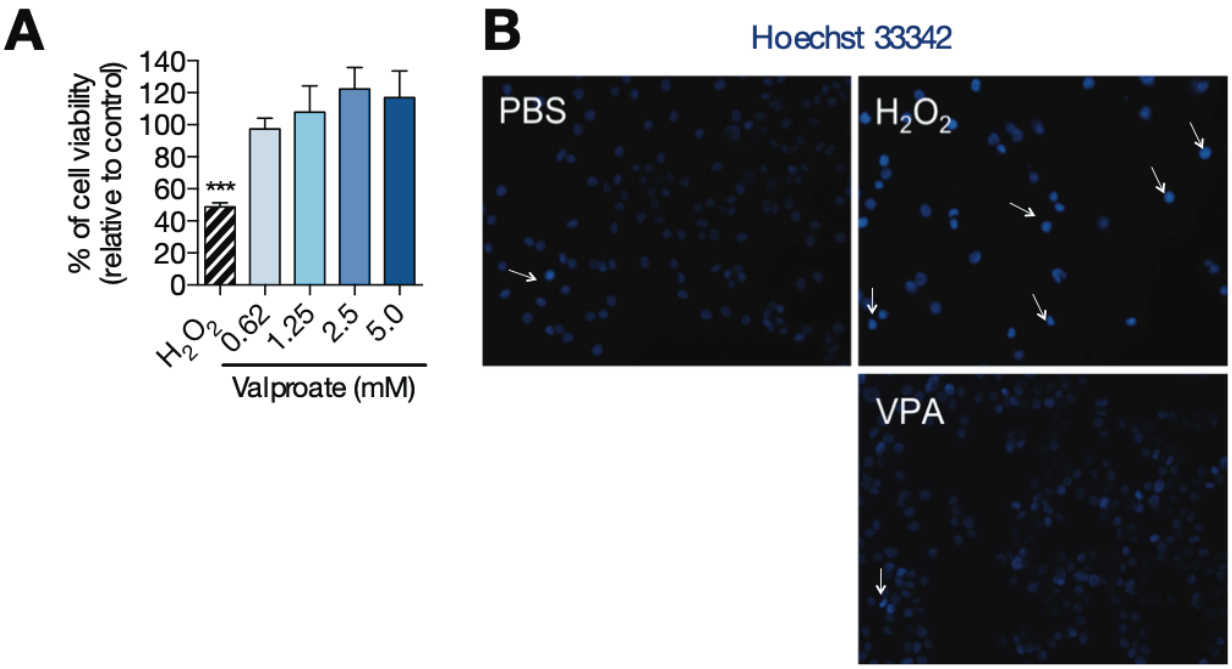

Figure S1. Survival of PC12 cells upon VPA treatment for 24h. PC12 cells were incubated with a concentration range of VPA (0.62 – 5 mM) for 24 h, and **(A)** MTT and **(B)** Hoechst 33342 labeling assays were performed. PC12 cells treated with the highest dose of VPA did not exhibit nuclear aggregation associated with apoptosis (white arrows) (n = 3/group). Data are means ± SEM. Significant differences were determined by one-way ANOVA (A) or Student t-tests (B).

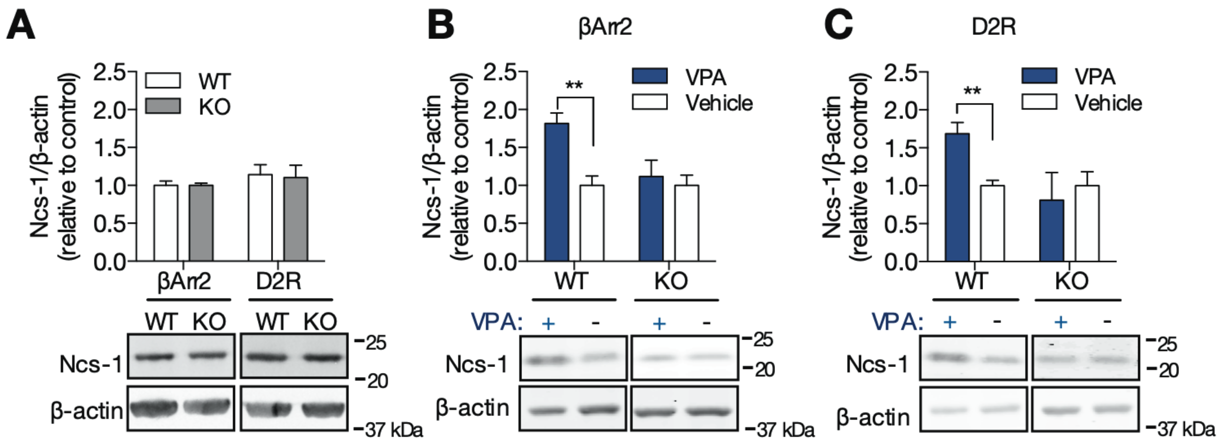

Figure S2. Chronic valproate treatment depends on βArr2 and D2R to induce Ncs-1 protein expression. (A) Western blot quantification of Ncs-1 protein expression in frontal cortex of βArr2 (left) and D2R (right) WT/KO (wild type/knockout) mice (n = 5/group). The bottom panel shows the representative western blots cropped from the same membrane obtained from this experiment. **(B-C)** Western blot quantification of Ncs-1 protein expression in βArr2 **(B)** or D2R **(C)** WT/KO mice treated chronically with VPA (25 g of drug per 1 kg of chow for 21 days) (n = 5/group). The bottom panel shows the representative western blots cropped from the same membrane obtained from this experiment. In contrast to WT mice, VPA-induced Ncs-1 protein expression is abolished in either βArr2 or D2R KO mice. In all cases data are represented as mean ± SEM. Western blot quantifications were normalized against β-actin and plotted relative to vehicle-treated samples, which was set to 1. Significant differences have been determined by Student's t-tests.

A

```
>gi|568815589|ref|NC_000009.12|:130170478-130172578 Homo sapiens
chromosome 9, GRCh38.p12 Primary Assembly

-2101 GAATGCAGTG GCACAATGAC GGCTCACTGC AGCCTCGCCC TGCTGGGTTC AAGTGATCCT
-2041 TCCACTTCAG CCACCCGAGT AACGGGGAGT TAGGCATGTG TCACCAAGCC CAGCTAACTT
                         F-2005 bp PRIMER
                      >>>>>>>>>>>>>>>>>>>>>>
-1981 TTAAAAATTT TTGTAGAGAT GAGGTCTCCC CTGTCAGGCT GGTCTTGAAC TCCTAAGCTC
                                           c-Rel
-1921 AAGTAATCCT TCCACCTCAG CCTCTCAAAG TGCTGGGATT ACCAGCATGA GCCACTGCAC
                        TATA signal
-1861 CCAGCCTGAG CACCTGGGTT TCTAAAGGGC TTTAACTACT ACTGCTGTCA TCCAGCCAGG
-1801 CCCTGGTTCC CGTGCTGGCT CTCCCTCCTC CCAGGCAGGA TGCTCAGCAC AGTCTGGTCA
-1741 ACTGTTAGCC CCCCACTGTG GGCCCCTCAC TGCTGTCCAC TCTGAGTTGG TCCTCAAGGT
-1681 TGGGACATTC TGCTCCCAGG TCTACACCAG ATCCCTCCAA CTGGGCCCAC CTCCTCTGCA
                                                      c-Ets
-1621 GAATGAGCTC CCCAAGCCAG CAAGTCCATC CCCATCTGAA GACTTCCTGG GGCCTGGGGC
                                                  TATA signal
-1561 TCAGCCTGGG CAGGGGCTTC AGGGGAAAGC AGGAGTGGAT GGAGTATGCT TAGAGGCAGG
-1501 AGAGATATTT GGGAGCTGGA AGGCACTCGG CCCTGCTCAG GGTTCCTCTG AGCTTTCTGG
                          AP3                                    AP-1
-1441 ACCCTCCGAG GTTAGAACAC TGTGGAAAGA AGGGAGAAAG AGACCCCACT CCTGCCTCAT
                                 AP-1
-1381 CCCTGCTAGC CCCGGGGAGG GCTGCCTCAT CCTCCCCGCT CTCTCCCCTA CCCCAGGGCC
-1321 CCAGCACCGG GTGTCATTCT TTCCTCCGTC CTTCGCTCAT TCACAGAGAT TTGCTGGGTA
-1261 ACACCTCAGG CGGCCTTCTG GGTTATGGAG ATGCCTCTGC CCTCTGGGAG GGTGTTCTCC
                    c-Ets                 Creb
-1201 CATGGAAGAG ACAAACAGGA TGCAGAAAAC ATAGGTGACG TTTGTGATGG ATGTGAAGGA
-1141 GAGAAACTAA AGCCGAAAAG GATAGGAGAG TTCTCAAGAT ATGATGATAT GTTGGATGAG
-1081 GTGGACCGGG AAGGCCATGC TGGACCTACC ACCTGAACCA CACCCCCCTA CGCGAATCCA
                  F-1048 bp PRIMER
              >>>>>>>>>>>>>>>>>>>>>>>>
-1021 CTGGCCTCTG TCCATTCAGG TGGAGCCTGT CACCCCAGCC TCGGCTCAGA GAAACAGCTC
                                          AP1                 Ets
-0961 TGAGCAGCTC TGACCTGTCA CTCCAGCAGC TCTGACCCAT CACTCTCTGC TGAAGTCCTT
-0901 TCATGGGCGC TGGTGGGCAG GGGCCCAGCT CCTCGGCGAG GGGCCTCTCC TGGCCTGCCC
-0841 CGTGGGCCCT GCCCTTCTCC CAGGTCCCCT GTGCCTGCAG CCGTGCGCAA GCCCGCAAGC
-0781 CGGGGAACTC TCCCTCGGGC ATGCAGGCCT CCCCGCCTCT CCAGGTTGCC TGGGCCAGAA
-0721 CGCTTCTCCA TCCACCTCCC TTCCTGACCC TGGGTTTGGA GGTCTCCTGC CCCCAGGCCG
-0661 AGAGTAGGTG CTCCCACCGC TACCTGCCGC ATCCCGCACA GCCCCCTGCC CGCCCTGCGA
                                                        F-588 bp PRIMER
                                                               >>>>>>>
-0601 GGGCTGGGGC ACTCCTGTCC CGTGGGCCGC CGTGTCCCCG GCACCCCATG AATCCGTGTC
      >>>>>>>>>>>>>>
         Oct-1/2                                                Ets
-0541 GAATGAATGA ATGTCTCCGG GCCGGGACCT GGCCTCATTC ACATCCGATC TTCCGGGCCC
-0481 TCCCCGCCGC GGCGGGGGCC GGAGCGCCAG GGCTGGATTC GGGGAGGGGG TGGCGGGGGT
-0421 GGCTGCCCCG CCCTGGGGCC GCGTTGAGCC GAGGGGACGC CCCTCCGGCT GGCTGGCGAG
-0361 GCCGGGGTCC AGCTGGCGGA GGCCGCGGCC AGGCGGGTGC GGGGCGGGAG GTGGCGCCGG
-0301 GCGCTGAGTG GCGGCTCCGG GTCCACCCCT GGCGCGCGGG TGCGGGCGGG AGGGGGGAGG
-0241 CGGGAGGCGG GGAGCGCAGG GGCGGCGGCA GGCGGGAGGC AGCAGCGAGC ATGTGCCGCG
-0181 GAGCCCAGTA ACCAGGGACG ACCGCGGCCA CACCGCGCCG GCGCCGGCGC CCAGCCCAGG
-0121 CAGCCCCGCG CCGCGGCGCC CGGACCGCCC GGGCCTGCCC AGCGGCCGCC CCACGCCCCG
-0061 GGCGCCCCGG CGCCGACAGC CGCGCAGCGC AGCGCGGGCG CCGCAGACAA AGGCGCGGCC
                                        R-PRIMER
      >>>TSS(NCS-1)               <<<<<<<<<<<<<<<<<<
+0001 CCGGCCCGGC CCGCCCGGCC CAGCCGCTCC TGCTGGGCGC CCCAACCGGG TCCGGCCCGG
+0061 GGGGGCGGGG GCCGCGGCCG CCGAGGATGG GGAAATCCAA CAGCAAGTTG AAGCCCGAAG
+0121 TTGTGGAGGA GCTGACCAGG AAGACCTACT GTGAGTGCTC CCAGCCCCCA GCCCGCGCCC
```

B

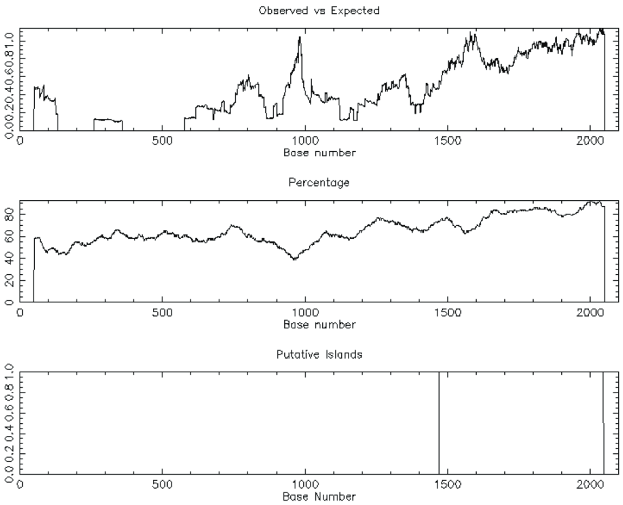

Figure S3. Upstream sequence of the human NCS-1 gene. (A) The 2,101-bp region immediately upstream from the *NCS-1* transcription start site (local 130,170,478-130,172,578) contains a number of predicted transcription factor binding sites and identifiable TATA box (underlined nucleotides in bold). c-Rel: REL proto-oncogene, NF-KB subunit; c-Ets: ETS proto-oncogene 1, transcription factor; C/EBP: CCAAT enhancer binding protein; AP-1: Jun proto-oncogene transcription factor; Creb: CAMP responsive element binding protein; oct1/2: POU class 2 homeobox. The highlighted sequence in yellow represents part of the *NCS-1* gene exon 1. Green and red nucleotides correspond to the untranslated region (UTR) and the coding region of the *NCS-1* gene, respectively. The 2,005 bp promoter insert was isolated by using the F-2005 and the R primers. Shorter inserts were obtained with F-1048/R and F-588/R primers. **(B)** GpC-rich region at the 5' end of the *NCS-1* gene (scanned with the EMBOSS CpGPlot at https://www.ebi.ac.uk/Tools/seqstats/emboss_cpgplot/).

UNEDITED GELS OF WESTERN BLOT USED IN THE PUBLICATION

Figure 1b

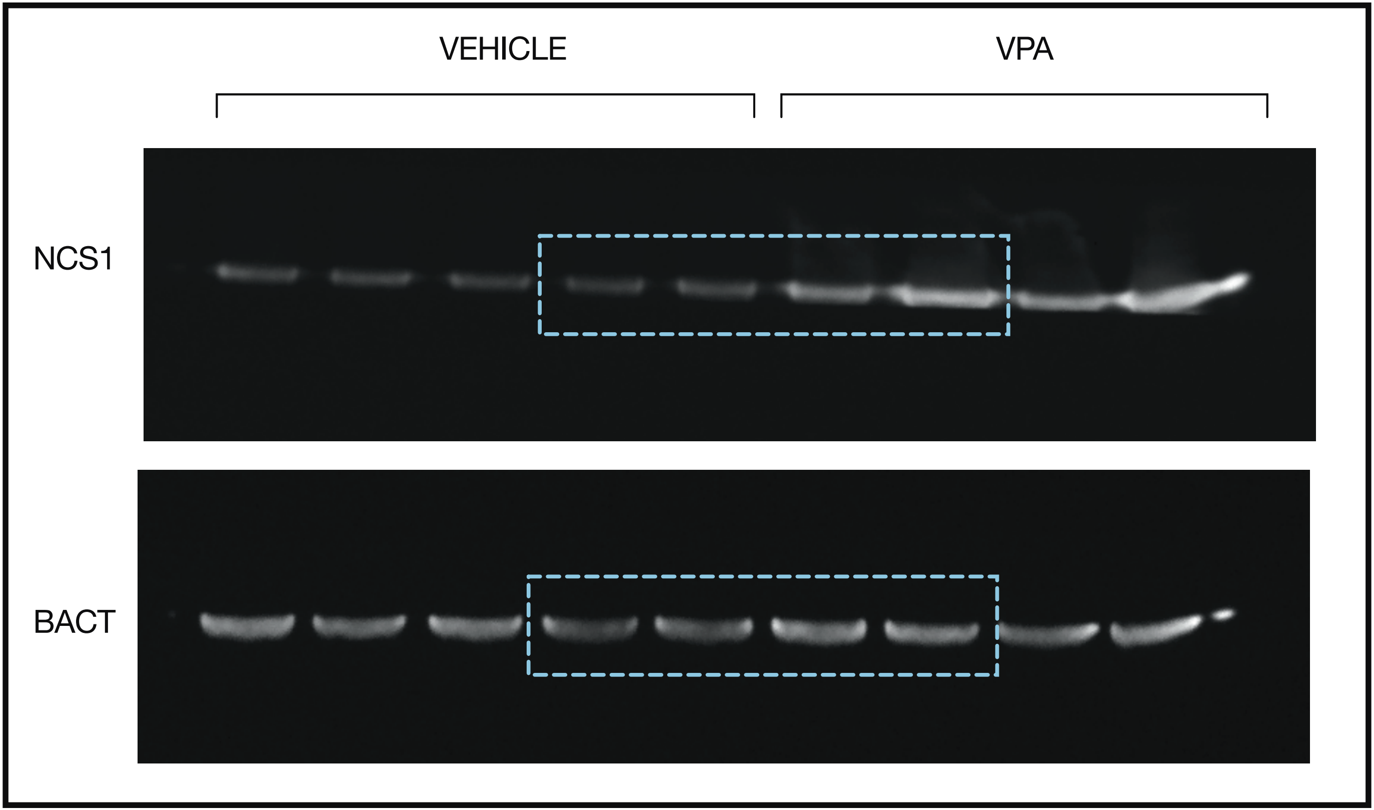

Figure 1c

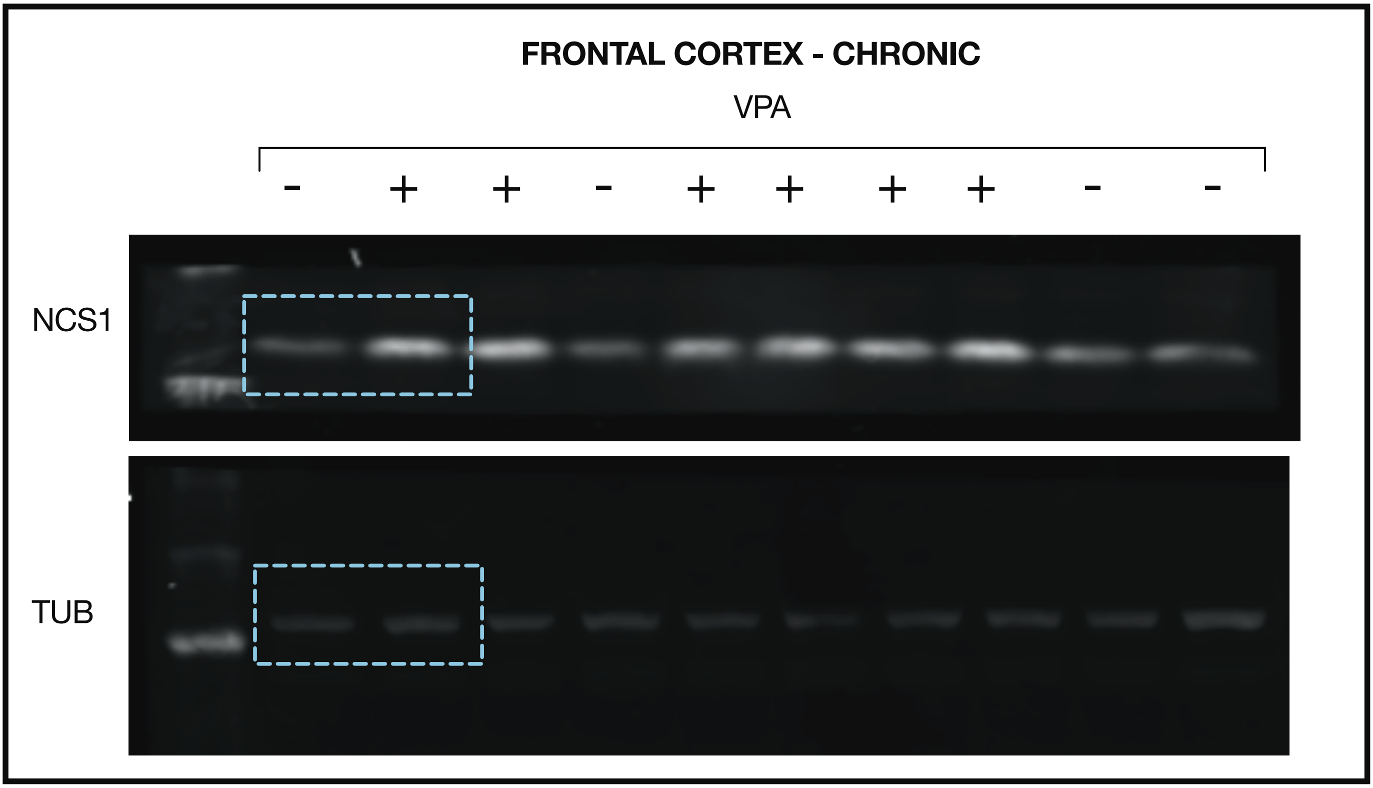

Figure 1c

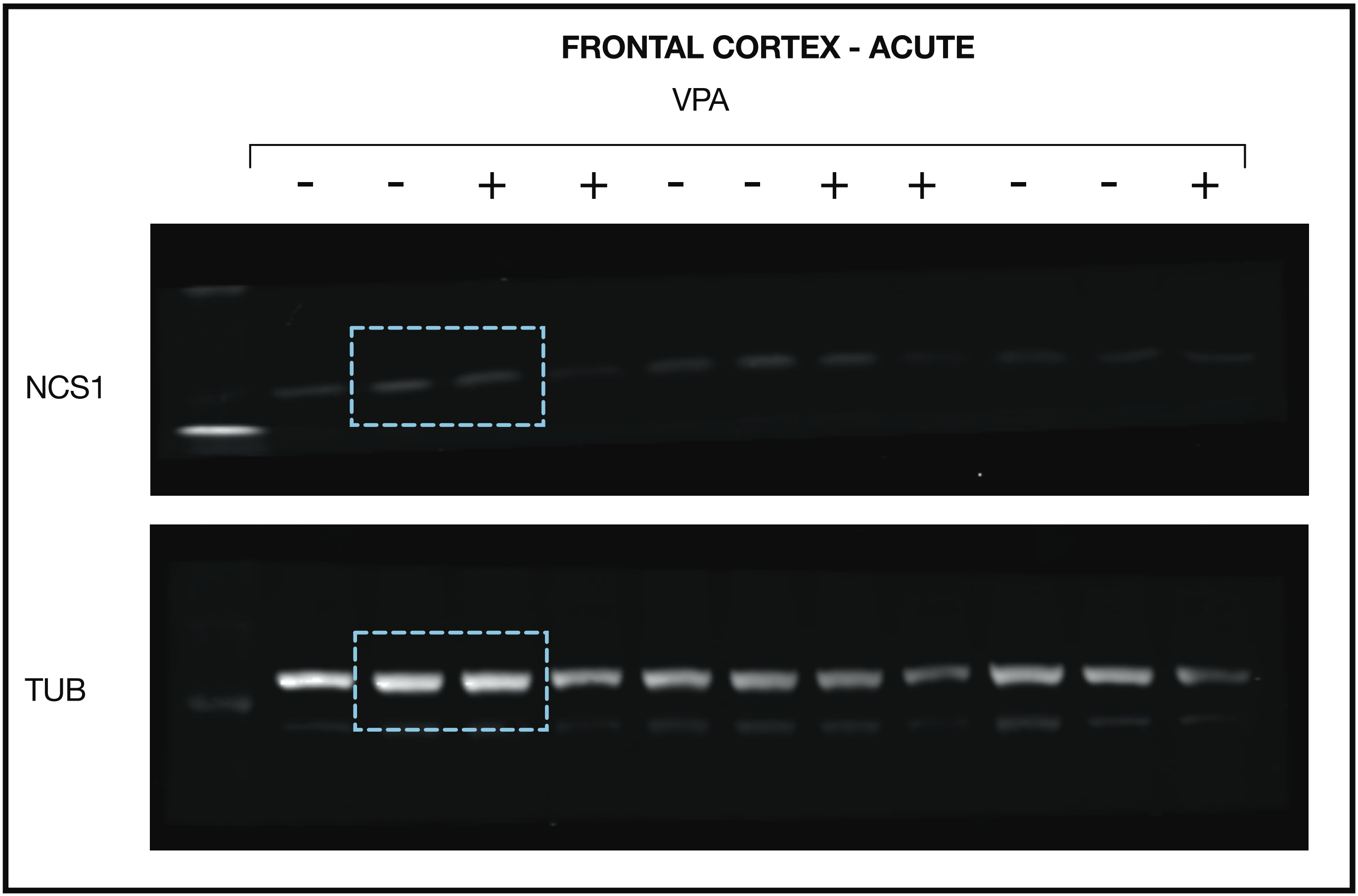

Figure 1c

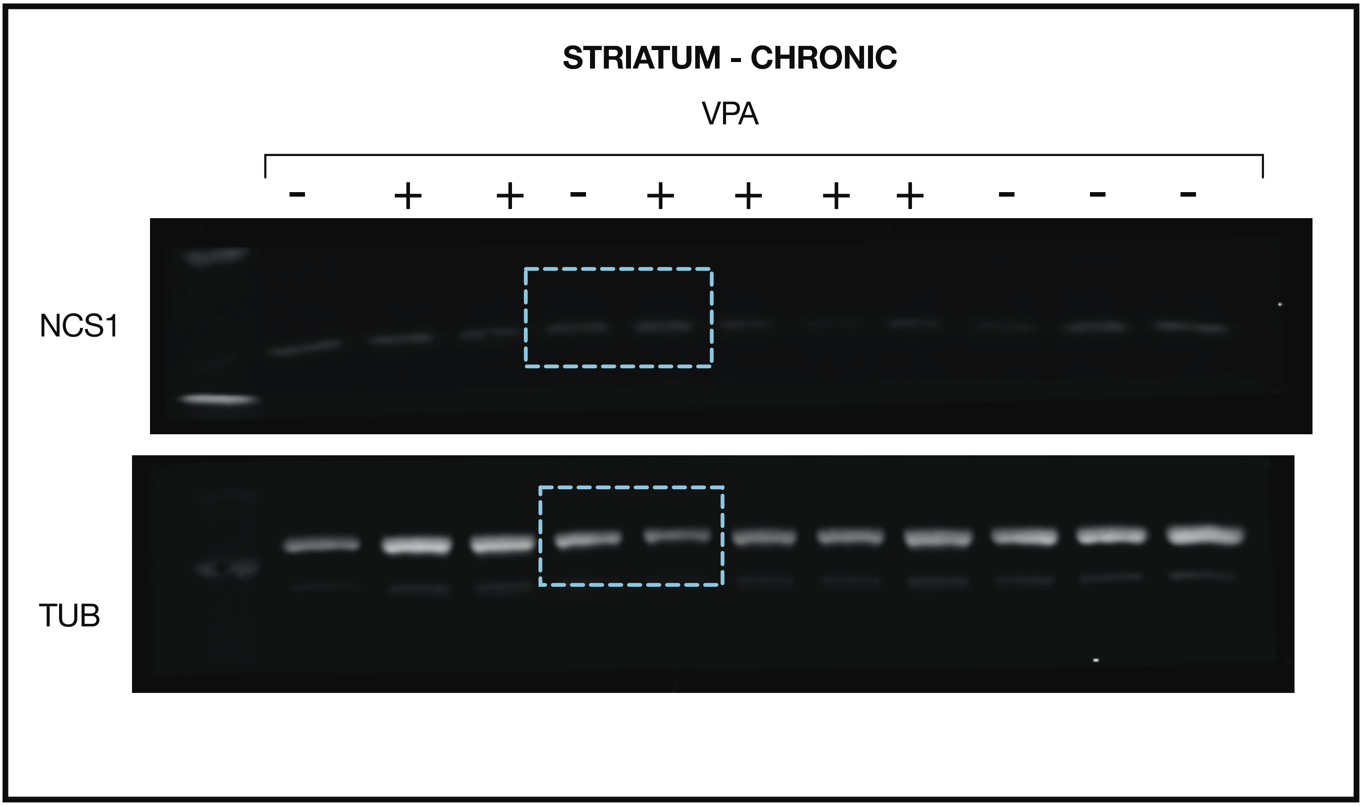

Figure 1c

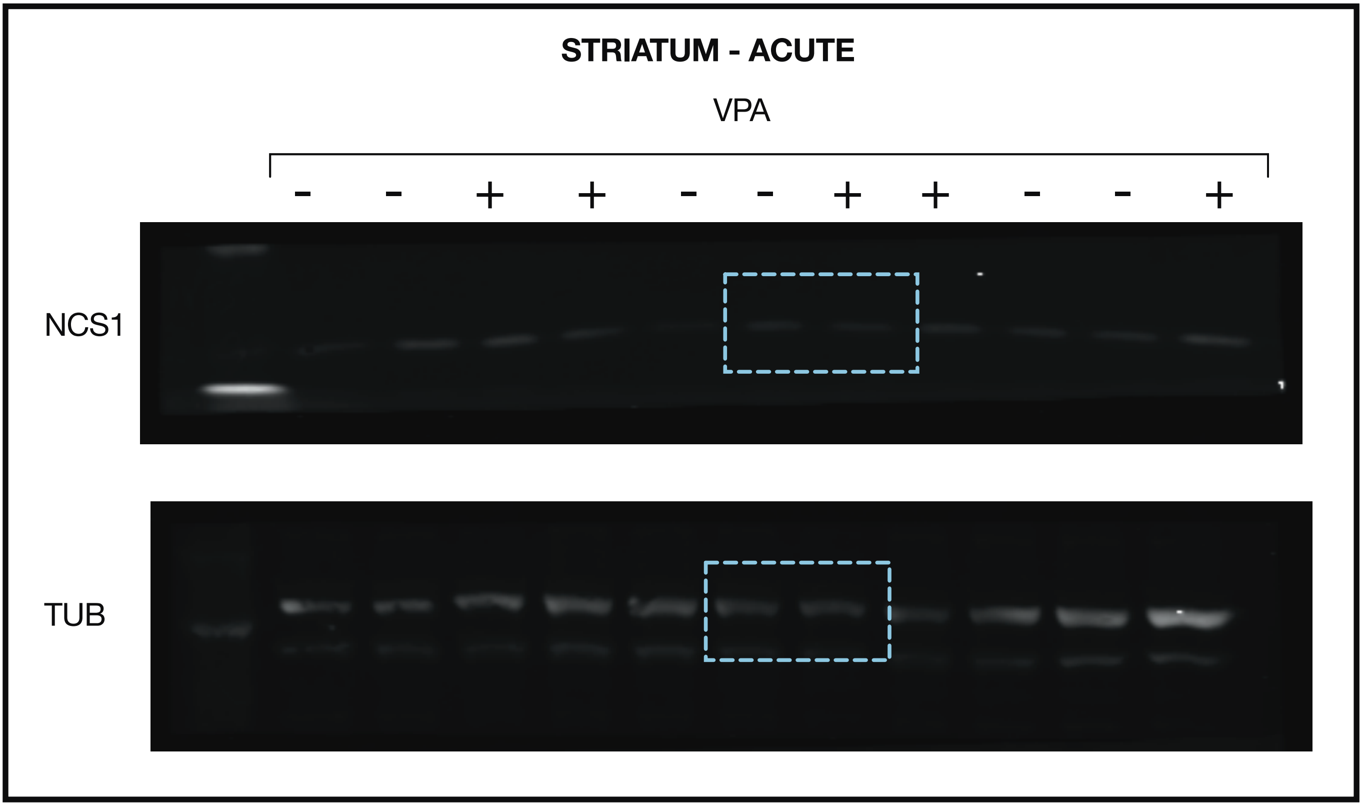

Figure 2b

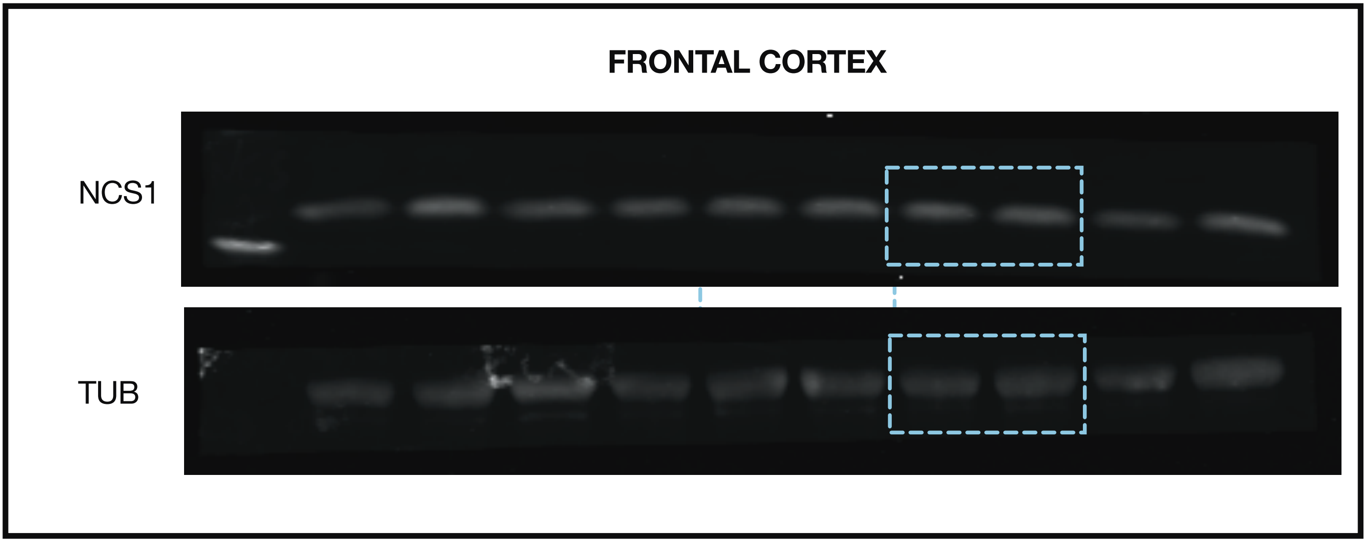

Figure 2b

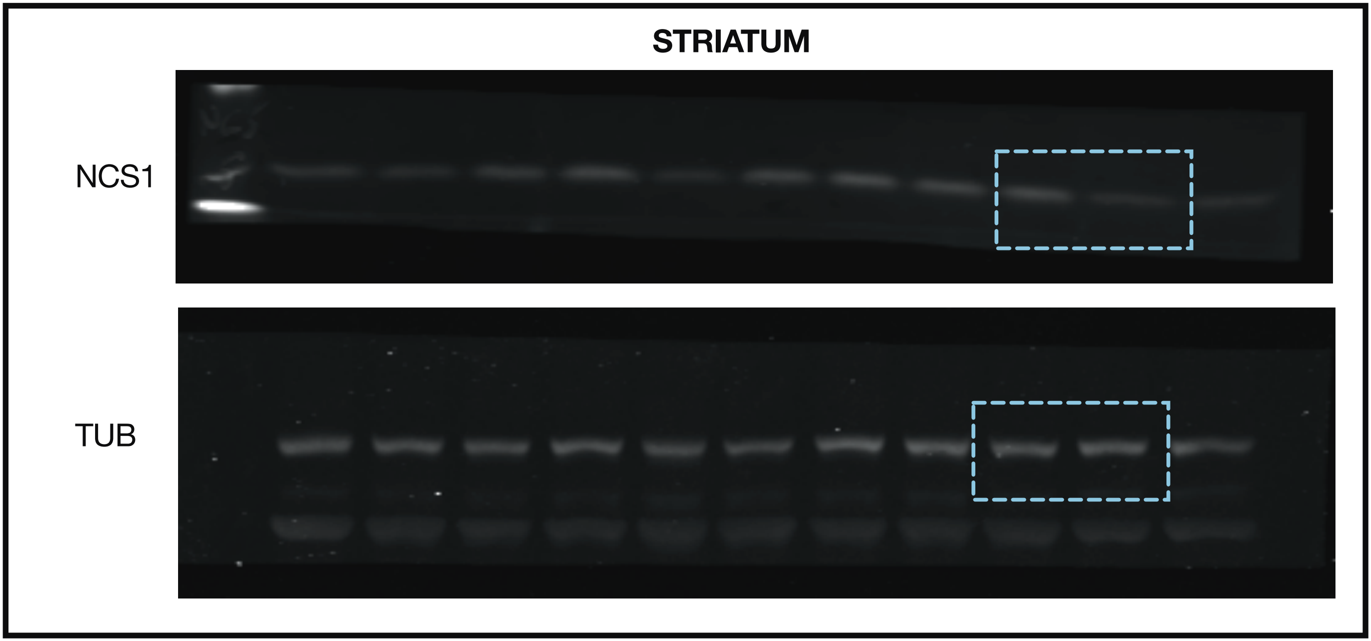

Figure 3b

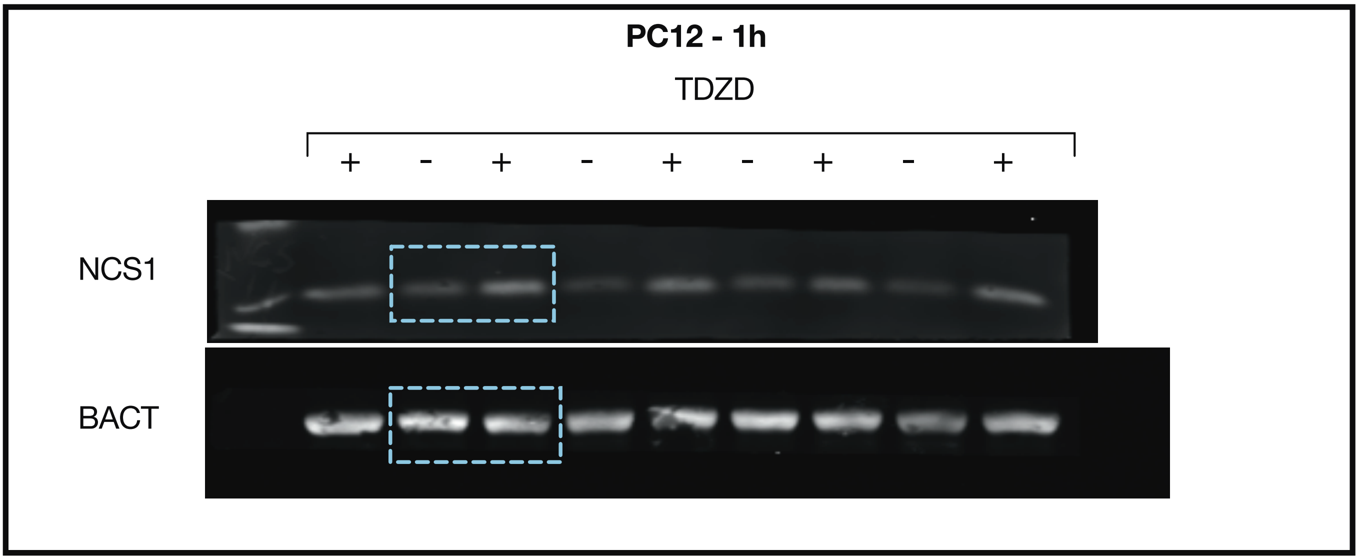

Figure 3b

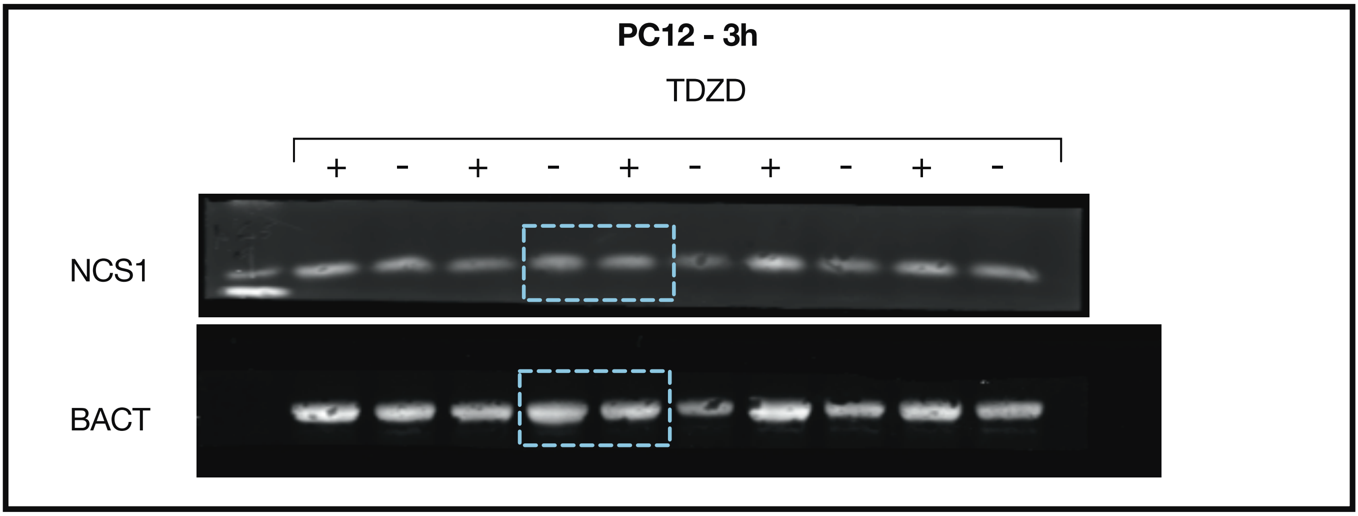

Figure 3b

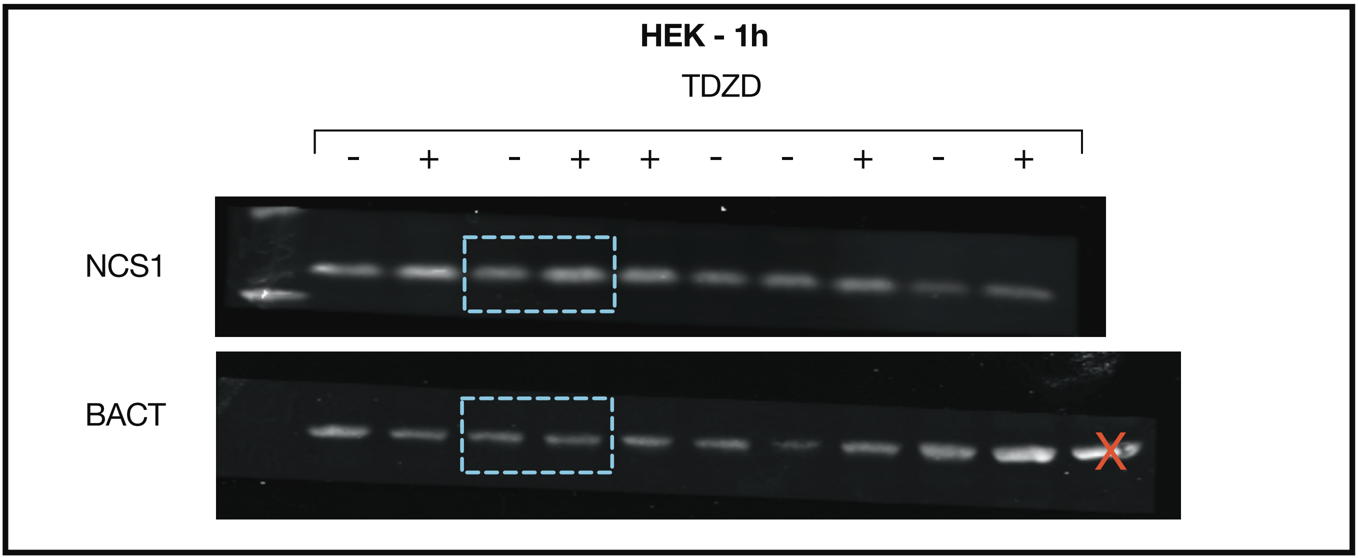

Figure 3c

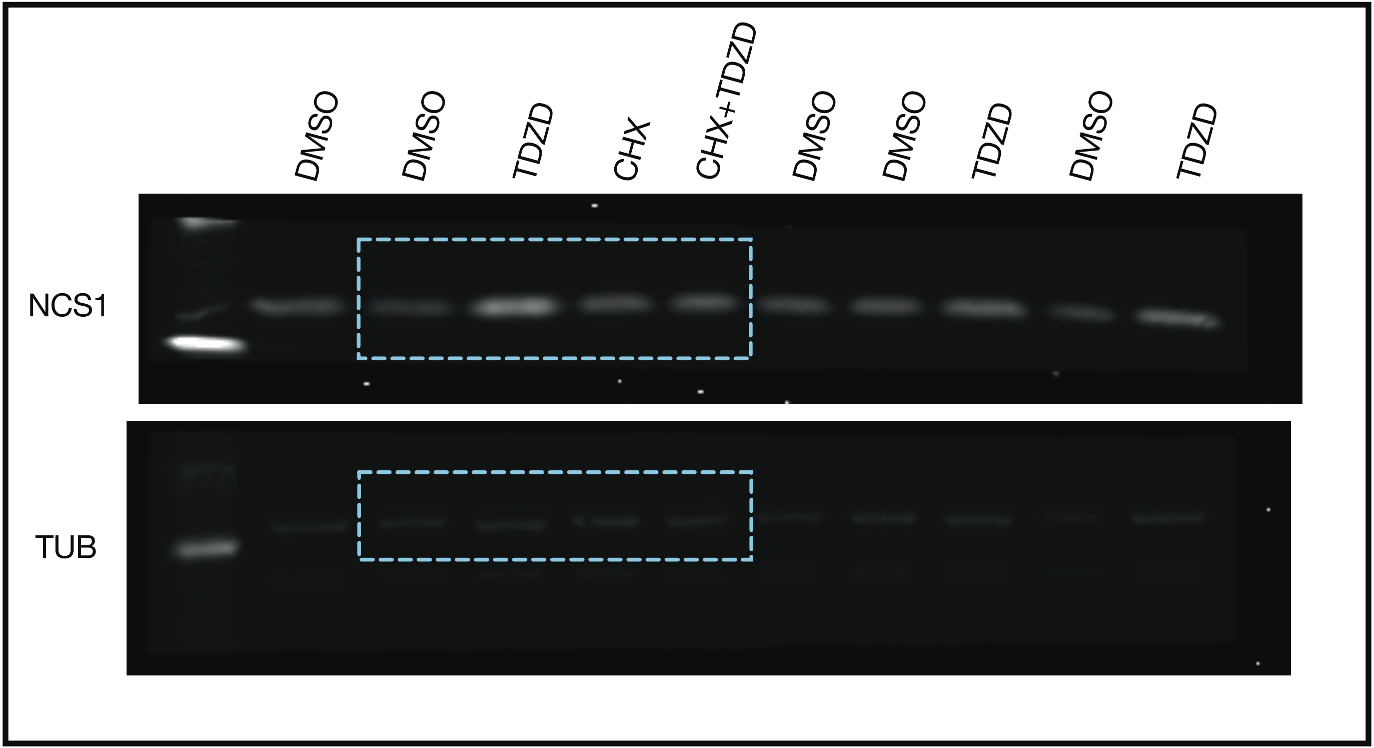

Figure 3d

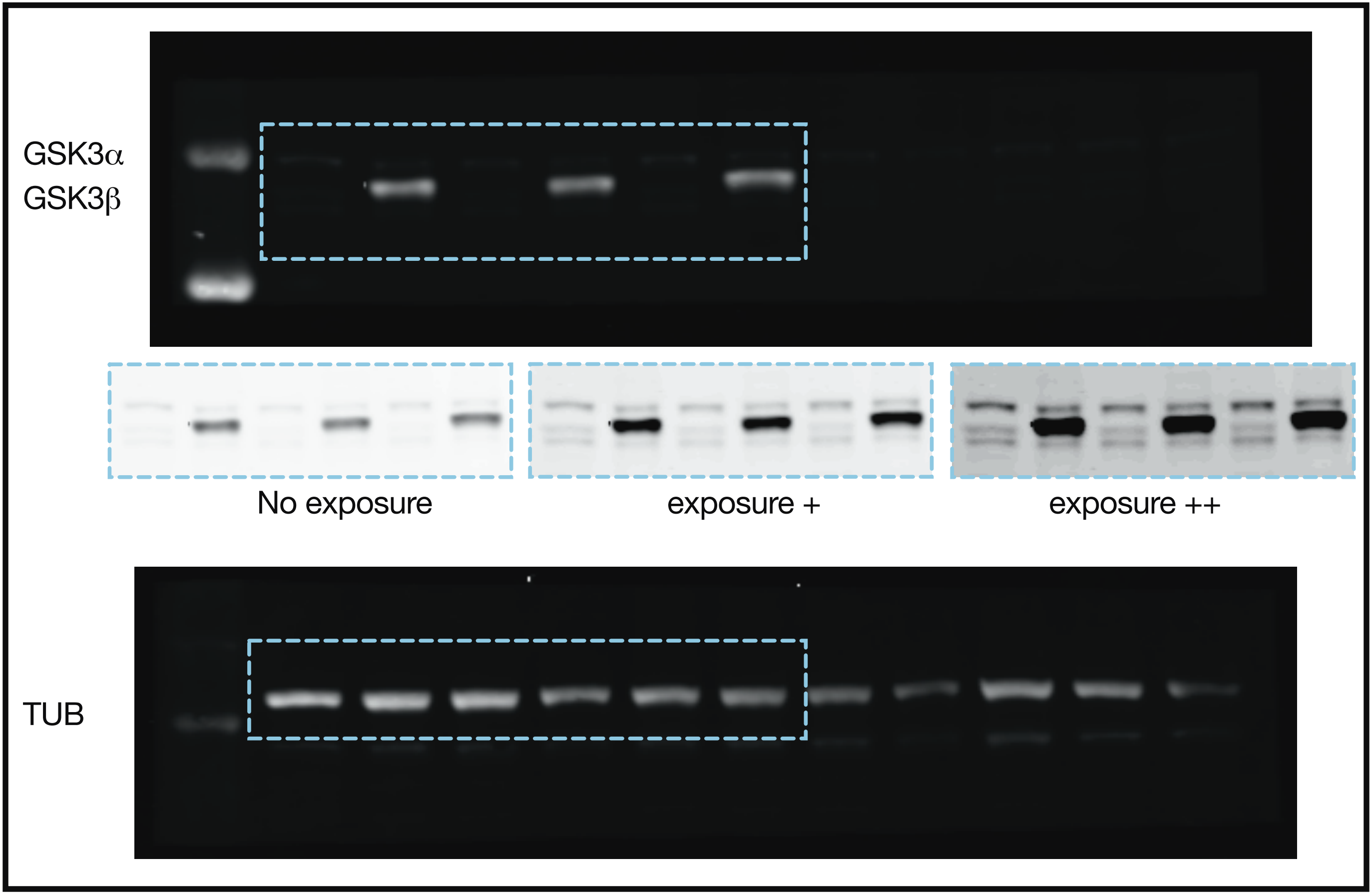

Figure 3f

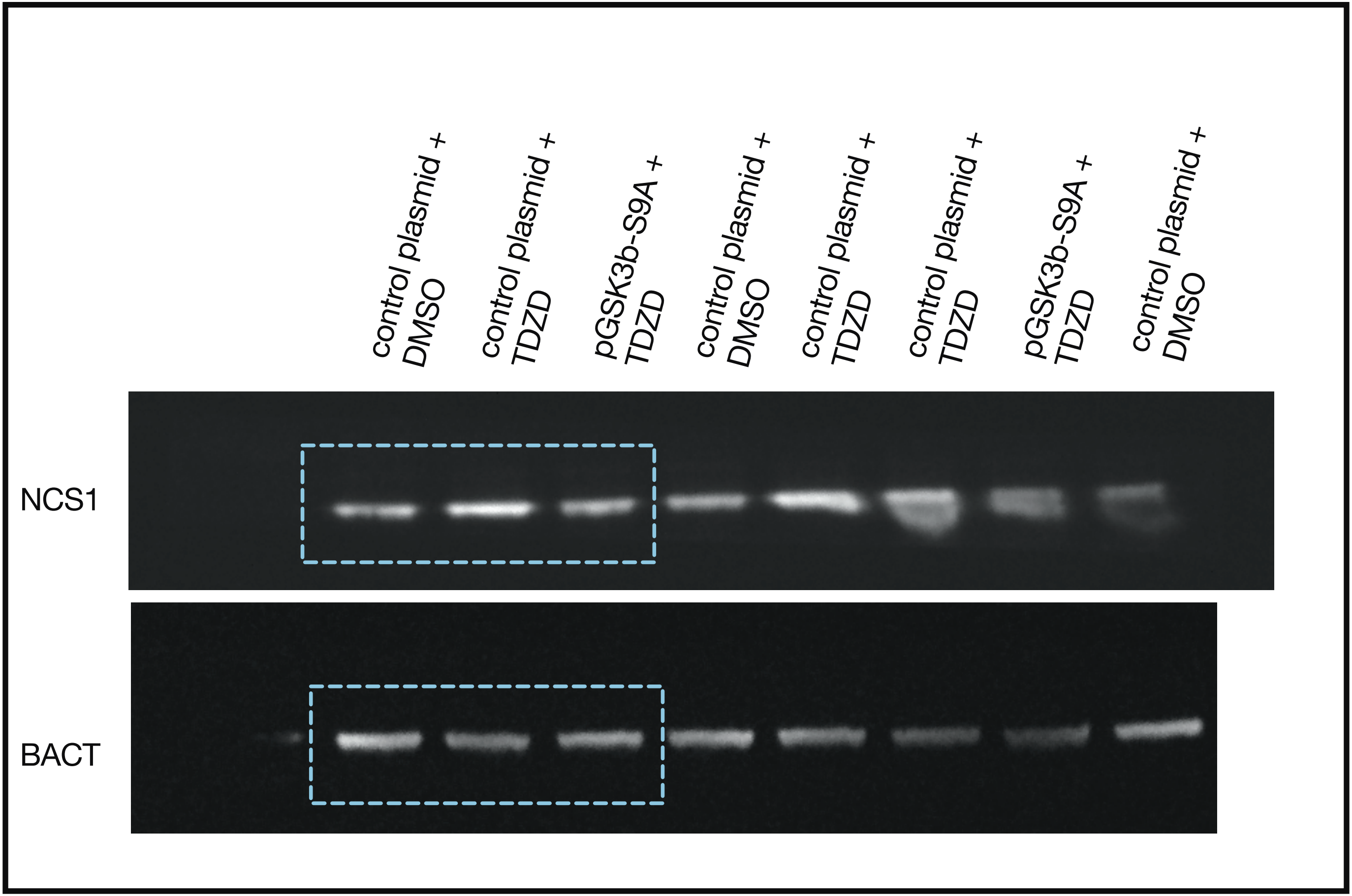
control plasmid + DMSO
control plasmid + TDZD
pGSK3b-S9A + TDZD
control plasmid + DMSO
control plasmid + TDZD
control plasmid + TDZD
pGSK3b-S9A + TDZD
control plasmid + DMSO
NCS1
BACT

Figure 3g

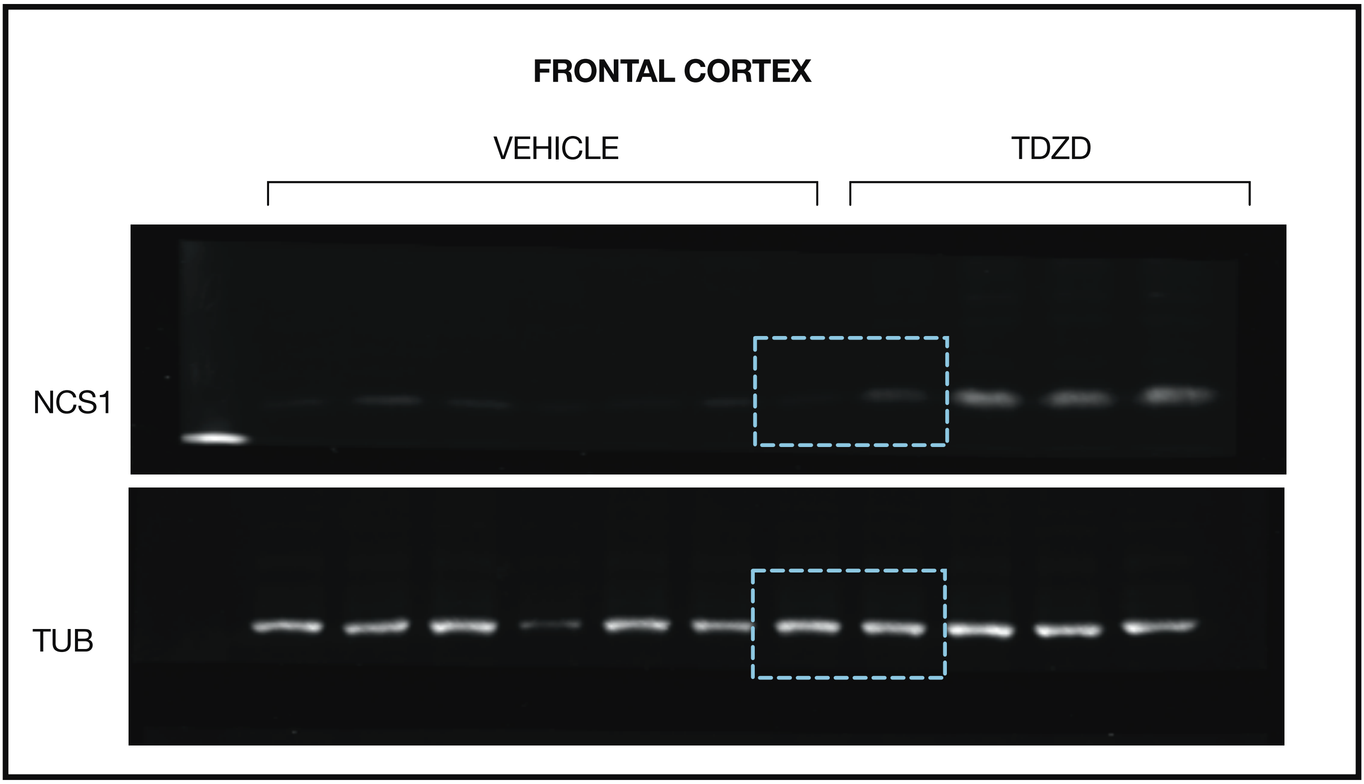

Figure 3g

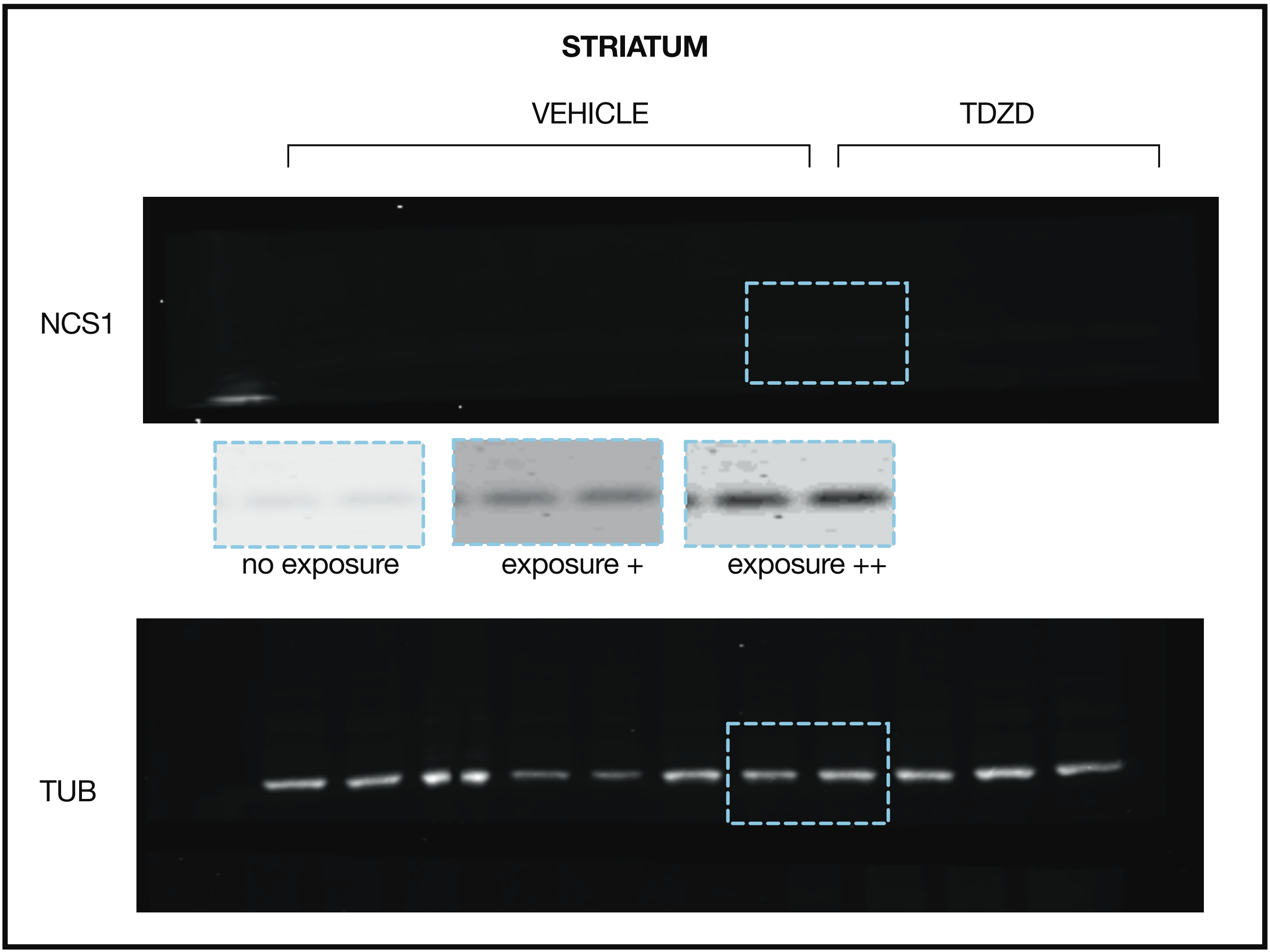

Figure 3h

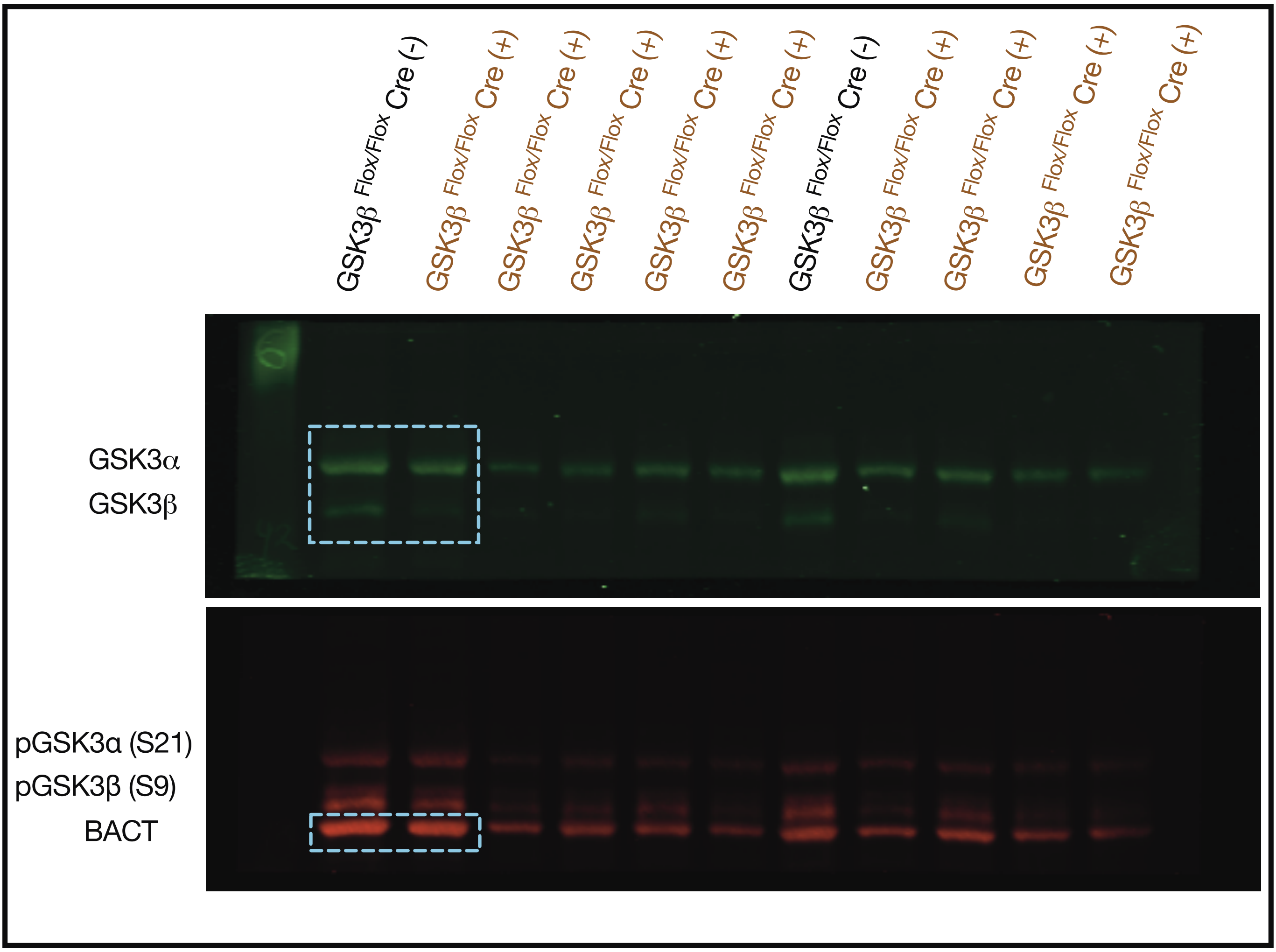

Figure 3h

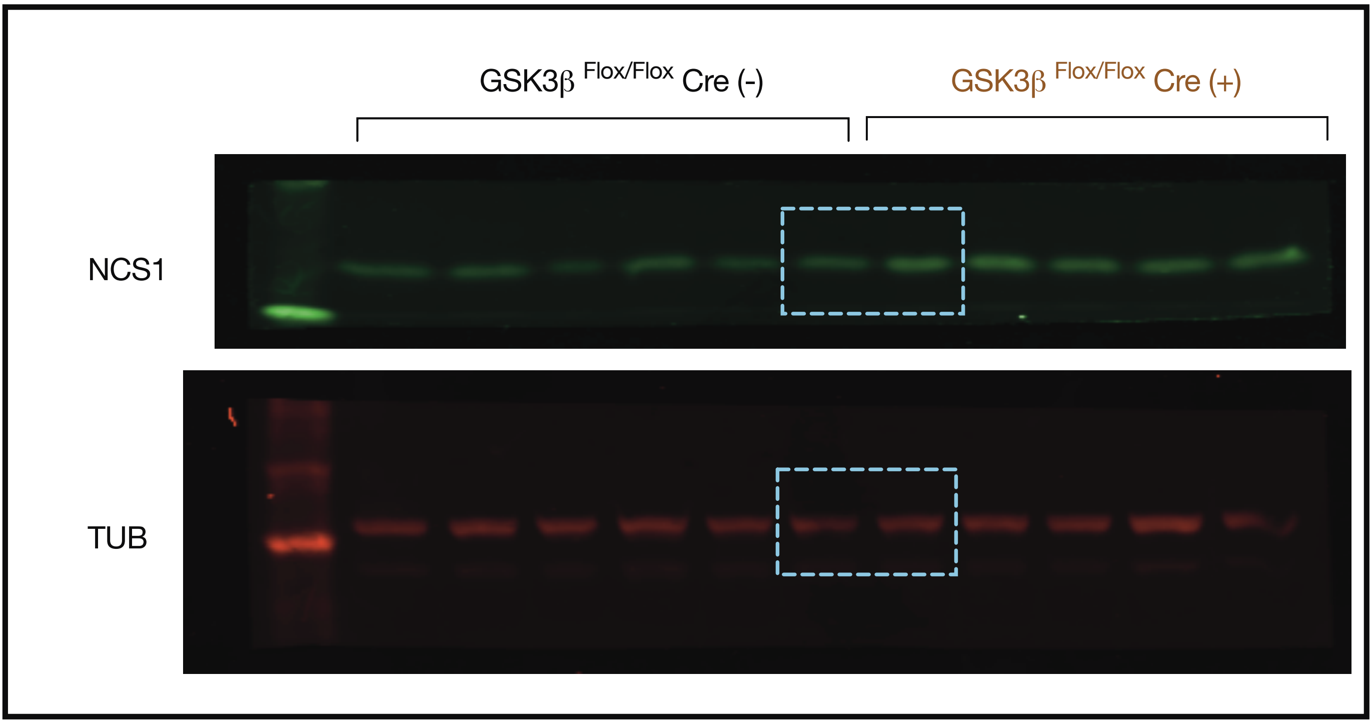

Figure 4a

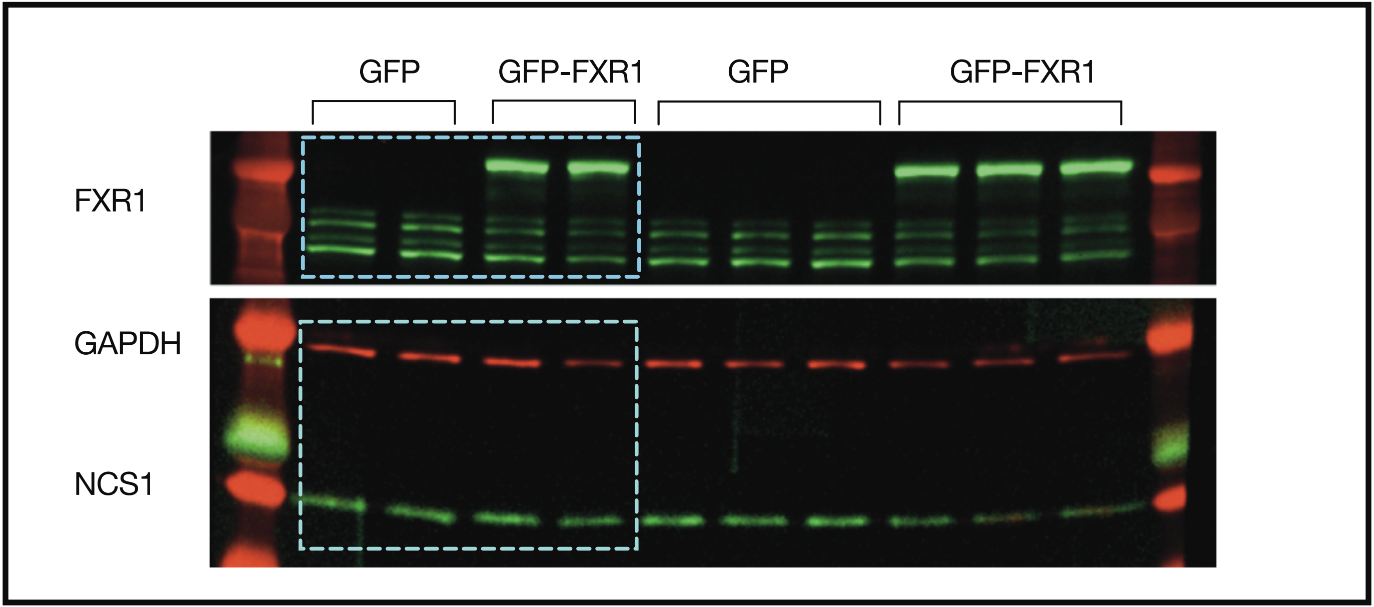

Figure 4B

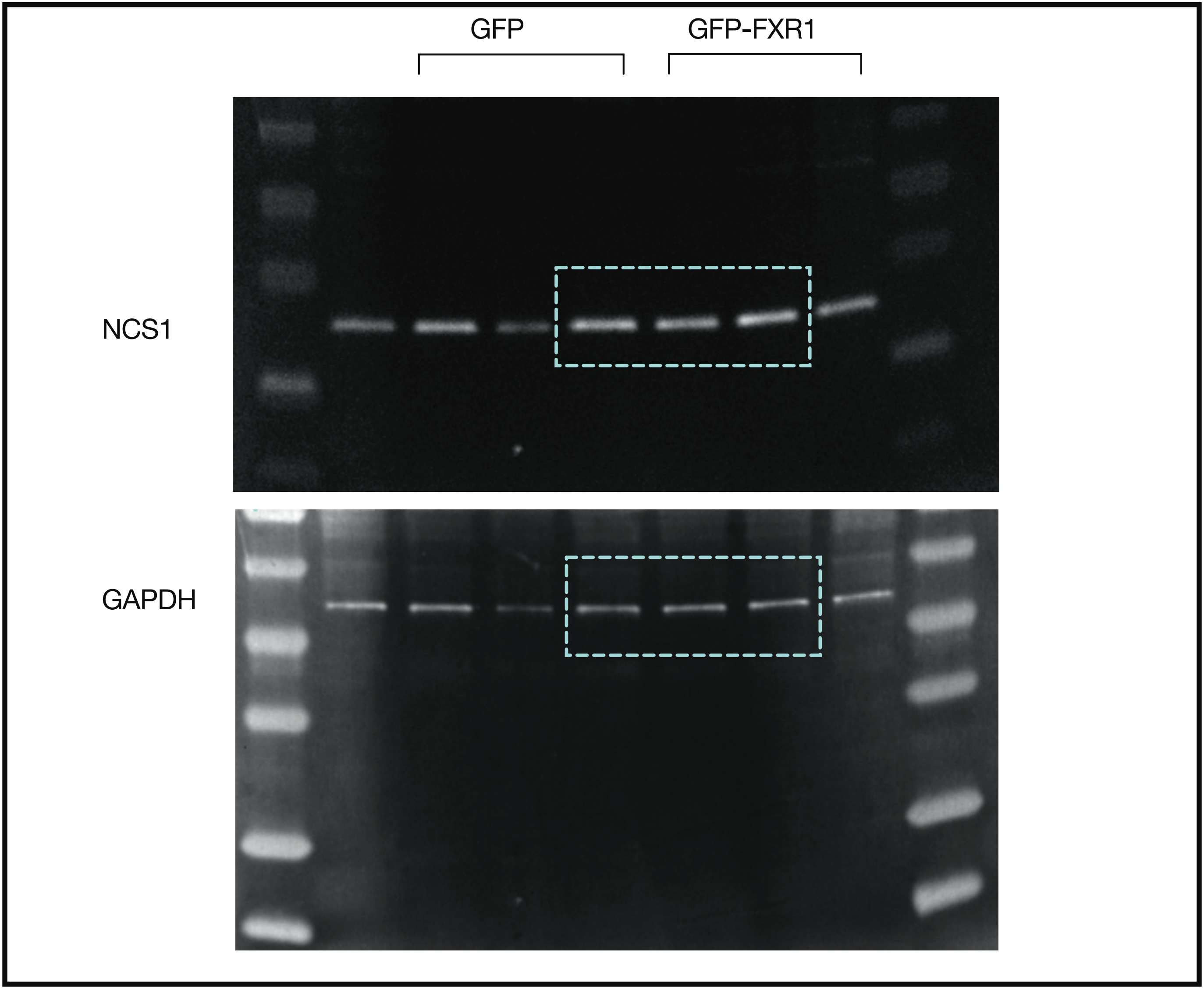

Figure 5c

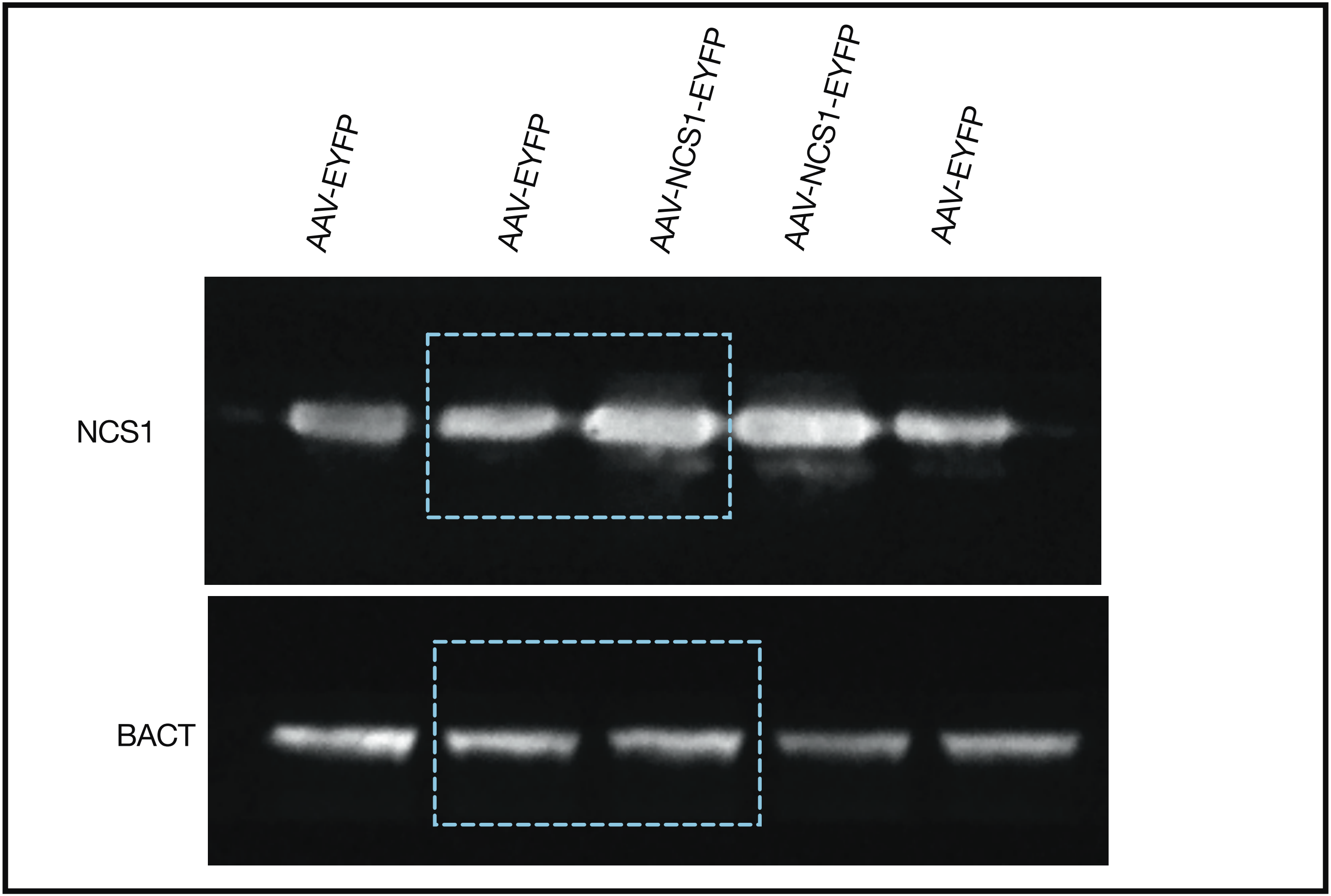

AAV-EYFP
AAV-EYFP
AAV-NCS1-EYFP
AAV-NCS1-EYFP
AAV-EYFP
NCS1
BACT

Figure S2a

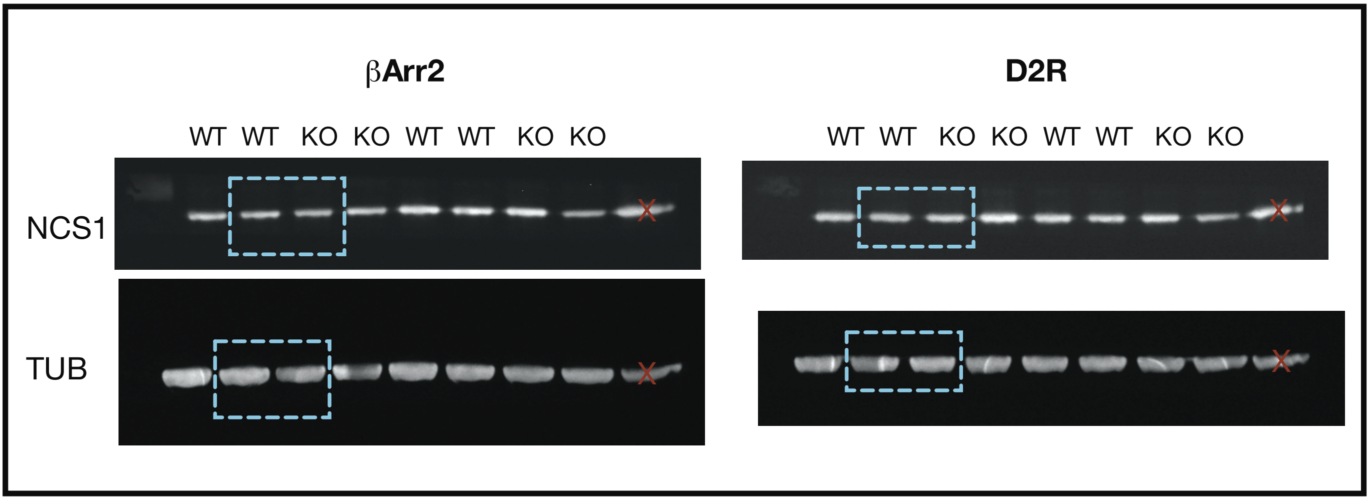

Figure S2b

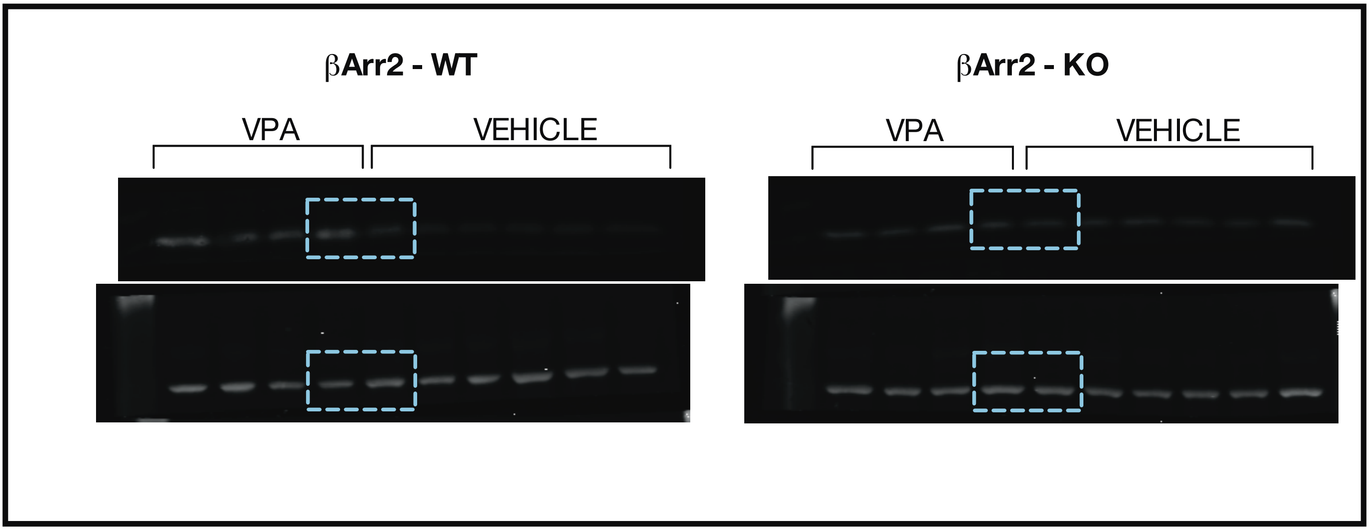

Figure S2c

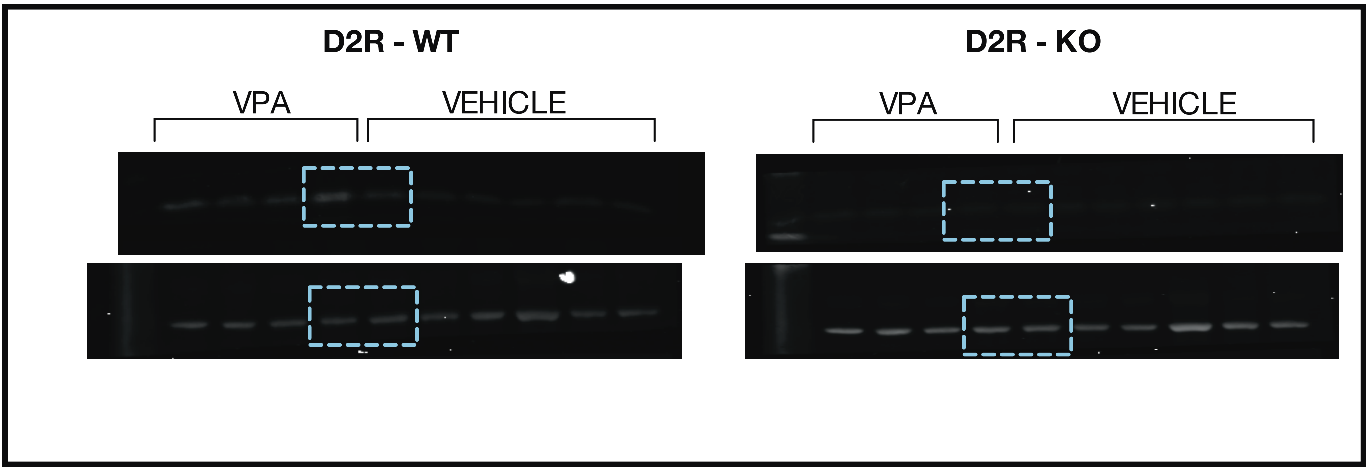

Supplement: Supplementary file 1 — Supplementary Information. [file 41598_2020_61248_MOESM1_ESM.pdf]
